# Prevalence and patterns of multimorbidity in Australian baby boomers: the Busselton healthy ageing study

**DOI:** 10.1186/s12889-021-11578-y

**Published:** 2021-08-11

**Authors:** Michael L. Hunter, Matthew W. Knuiman, Bill (A.W.) Musk, Jennie Hui, Kevin Murray, John P. Beilby, David R. Hillman, Joseph Hung, Robert U. Newton, Romola S. Bucks, Leon Straker, John P. Walsh, Kun Zhu, David G. Bruce, Robert H. Eikelboom, Timothy M. E. Davis, David A. Mackey, Alan L. James

**Affiliations:** 1 Busselton Population Medical Research Institute Inc, Nedlands, WA 6009 Australia; 2 School of Population and Global Health, The University of Western Australia, Crawley, WA 6009 Australia; 3 BPMRI Busselton Health Study Centre, PO Box 659, Busselton, Western Australia 6280; 4 PathWest Laboratory Medicine of WA, QEII Medical Centre, Nedlands, WA 6009 Australia; 5 Department of Pulmonary Physiology and Sleep Medicine, Sir Charles Gairdner Hospital, Nedlands, WA 6009 Australia; 6 Faculty of Health and Medical Sciences, Medical School, University of Western Australia, Crawley, 6009 Australia; 7 Exercise Medicine Research Institute, Edith Cowan University, Joondalup, WA 6027 Australia; 8 School of Psychological Science, University of Western Australia, Perth, WA 6083 Australia; 9 School of Physiotherapy and Exercise Science, Curtin University, Bentley, WA 6845 Australia; 10 Department of Endocrinology and Diabetes, Sir Charles Gairdner Hospital, Nedlands, WA 6009 Australia; 11 Ear Science Institute Australia, Subiaco, WA 6008 Australia; 12 Ear Sciences Centre, The University of Western Australia, Crawley, WA Australia; 13 Department of Speech Language Pathology and Audiology, University of Pretoria, Pretoria, South Africa; 14 Medical School, University of Western Australia, Fremantle Hospital, Fremantle, WA 6959 Australia; 15 Centre for Ophthalmology and Visual Science, University of Western Australia, Lions Eye Institute, Perth, Australia

**Keywords:** Multimorbidity, Ageing, Co-morbidities, Middle-aged, Chronic disease

## Abstract

**Background and objective:**

Chronic medical conditions accumulate within individuals with age. However, knowledge concerning the trends, patterns and determinants of multimorbidity remains limited. This study assessed the prevalence and patterns of multimorbidity using extensive individual phenotyping in a general population of Australian middle-aged adults.

**Methods:**

Participants (*n* = 5029, 55% female), born between 1946 and 1964 and attending the cross-sectional phase of the Busselton Healthy Ageing Study (BHAS) between 2010 and 2015, were studied. Prevalence of 21 chronic conditions was estimated using clinical measurement, validated instrument scores and/or self-reported doctor-diagnosis. Non-random patterns of multimorbidity were explored using observed/expected (O/E) prevalence ratios and latent class analysis (LCA). Variables associated with numbers of conditions and class of multimorbidity were investigated.

**Results:**

The individual prevalence of 21 chronic conditions ranged from 2 to 54% and multimorbidity was common with 73% of the cohort having 2 or more chronic conditions. (mean ± SD 2.75 ± 1.84, median = 2.00, range 0–13). The prevalence of multimorbidity increased with age, obesity, physical inactivity, tobacco smoking and family history of asthma, diabetes, myocardial infarct or cancer. There were 13 pairs and 27 triplets of conditions identified with a prevalence > 1.5% and O/E > 1.5. Of the triplets, arthritis (> 50%), bowel disease (> 33%) and depression-anxiety (> 33%) were observed most commonly. LCA modelling identified 4 statistically and clinically distinct classes of multimorbidity labelled as: 1) “Healthy” (70%) with average of 1.95 conditions; 2) “Respiratory and Atopy” (11%, 3.65 conditions); 3) “Non-cardiometabolic” (14%, 4.77 conditions), and 4) “Cardiometabolic” (5%, 6.32 conditions). Predictors of multimorbidity class membership differed between classes and differed from predictors of number of co-occurring conditions.

**Conclusion:**

Multimorbidity is common among middle-aged adults from a general population. Some conditions associated with ageing such as arthritis, bowel disease and depression-anxiety co-occur in clinically distinct patterns and at higher prevalence than expected by chance. These findings may inform further studies into shared biological and environmental causes of co-occurring conditions of ageing. Recognition of distinct patterns of multimorbidity may aid in a holistic approach to care management in individuals presenting with multiple chronic conditions, while also guiding health resource allocation in ageing populations.

**Supplementary Information:**

The online version contains supplementary material available at 10.1186/s12889-021-11578-y.

## Background

In Australia and other developed countries, increasing life expectancy and the transition of the Baby Boomer generation (adults born 1946 to 1964) from middle into older age has led to an increasing number of people with multimorbidity, commonly defined as the presence of two or more chronic, long-term health conditions [1]. Most studies report that multimorbidity is the norm in the elderly and common in middle-age (45–64 years) [1–3]. Multimorbidity is associated with greater risk of mortality, poor quality of life, psychological stress, cognitive decline, impaired functional capacity, greater use of health and social support services and reduced work productivity [3–10]. The growing prevalence of multimorbidity is also recognised in low- to middle income countries where additional factors such as the presence of communicable diseases, demographic transition and socio-economic challenges contribute to the complexity and burden of multimorbidity in these settings [11].

Individuals with multimorbidity have complex healthcare needs that may not be adequately addressed by models of health care centred on managing and treating single conditions. They are also at increased risk of adverse drug effects from polypharmacy and potential harm from conflicting treatment or lifestyle recommendations [12]. Despite attempts to address these complex needs in clinical guidelines [2, 13–15], knowledge concerning the trends, patterns and determinants of multimorbidity remains sub-optimal. This has significant implications for future healthcare service planning and allocation of resources.

There have been several studies of the prevalence of multimorbidity in primary care and general populations including in Australia over the last decade [16]. These have mostly involved cohorts, aged > 65+ years and have used clinical administrative data, self-report or national survey datasets to identify conditions and a variety of measures to define multimorbidity [16–23]. The measures of multimorbidity in these studies are constrained by the available data and vary widely in terms of the number of considered conditions and their definitions [24]. As a result, the prevalence and patterns of multimorbidity derived from different data sources are often inconsistent, even when generated from the same population sample [25, 26]. Notably, there have been few community-based studies of multimorbidity, particularly in middle-aged adults, that have used clinically-defined, objective measures of chronic disease [14]. In their review Fortin et al. concluded that, in general, multimorbidity increases with age, especially from the age of 40 years with a plateau around 70 years, if > 2 conditions are used to define multimorbidity [14]. This plateau was less evident if > 3 conditions was used to define multimorbidity.

Certain conditions may be more likely to cluster than others [27]. This may be the consequence of shared pathophysiological pathways, in which the presence of one condition increases the risk of another, or may reflect common environmental or biological risk factors. In order to better understand the factors underlying patterns of comorbidity and to thus inform healthcare planning, there have been recent calls for studies in general population samples, assessing a wide range of conditions characterised by objective clinical data including information on symptoms, lifestyle behaviours and environmental risk factors [28]. General population samples matching these requirements, particularly in middle-age when intervention for chronic conditions is likely to have greatest impact in older age, are few.

The Busselton Healthy Ageing Study (BHAS) is one such study where detailed measures of physical function, cognition, well-being, quality of life, medical history and biological samples have been collected enabling investigation into specific interactions between disease processes and symptoms within individuals [29]. The objective of this analysis was to investigate the prevalence and patterns of multimorbidity using a range of measures of chronic disease collected in the BHAS dataset. We hypothesised that co-occurring conditions are common in middle-aged adults, that there are identifiable non-random patterns of multimorbidity and that these patterns are associated with distinct risk factors and/or explanatory variables.

## Methods

### Population study sample

A comprehensive, community-based health survey of adults born 1946–1964 residing within the City of Busselton’s local government electoral boundary in Western Australia was undertaken from May 2010 to December 2015. All people listed on the compulsory Western Australian Electoral Commission Electoral Roll were invited to participate. Recruitment was via postal invitation and by telephone where a contact number was available. A total of 82% of those on the electoral register could be contacted and confirmed eligible (non-institutionalised and still living in the region) and 76% of those contacted (5107) participated in the study. Ethical approval for this study was obtained from the Human Research Ethics Committee of the University of Western Australia (RA/4/1/2203). All study participants provided written informed consent.

Participants completed a detailed questionnaire that included items relating to medical and family history, symptoms, physical activity and diet and nutrition (see Supplementary File 1). They attended the survey centre for up to 4 h of clinical assessments, which included: spirometry, skin prick (atopy), audiometry, cardiovascular profiling (Ankle-Brachial Index, ABI) and electrocardiogram (ECG), bone densitometry and an in-home overnight Apnealink™ sleep study. Participants also provided a fasting blood sample for blood glucose, haemoglobin and white cell count, glycated haemoglobin (HbA1c) and estimated glomerular filtration rate [30]. All clinical tests were conducted by accredited pathology laboratories (PathWest, Busselton & Nedlands, Western Australia). The complete BHAS protocol has been described in detail previously [29].

Based on the participants’ responses and testing, 21 conditions were defined and included in this analysis (Table 1). Six conditions were defined using only objective measures or validated instrument scale score [31–34]. Seven conditions were defined using a combination of objective measures or validated instrument *plus* self-reported doctor diagnosis and eight conditions were defined only by self-reported doctor diagnosis which was a “Yes” response to the question, “Has a Doctor ever diagnosed you with…?”. The list of conditions used in this analysis ensured that the condition was prevalent and chronic using the best available data from questionnaire responses or clinical assessment.

**Table 1:** Ranked prevalence of the 21 chronic conditions, with definitions, in 5029 Baby Boomers from the Busselton Healthy Ageing Study

Condition	Definition	Prevalence (%)
Atopy	Positive (wheal > 3 mm) on skin prick tests for common allergens (dust-mites, grasses, moulds, cat hair, dog dander)	54.4
Arthritis	Self-reported doctor-diagnosed	25.1
Bowel disease	Self-reported doctor-diagnosed colonic polyps, Crohn’s disease, ulcerative colitis, irritable bowel syndrome or diverticular disease	23.3
Liver disease	Self-reported doctor-diagnosed cirrhosis of the liver *or* ALT > 40 U/L in men or > 35 U/L in women	23.1
Depression/anxiety	Current treatment *or* PHQ9 or GAD7 scores indicative of current depression or anxiety, respectively	20.5
Bronchitis	Self-reported chronic cough or phlegm on most days for > 3 months each year	17.6
Eye condition	Self-reported macular degeneration, glaucoma, cataract, non-diabetes related serious eye condition resulting in loss of vision	14.6
Asthma	Self-reported doctor-diagnosed asthma	14.2
Heart disease	Self-reported doctor-diagnosed angina, cardiac pacemaker, myocardial infarction, coronary angioplasty or stent, or bypass surgery or Abnormal ECG	9.7
Thyroid disease	Self-reported doctor-diagnosed	8.6
Low back pain	Current self-reported chronic low back pain lasting > 3 months and Oswestry Disability Index > 12	8.5
Skin cancer	Self-reported doctor-diagnosed	8.2
Obstructive Sleep Apnoea	Apnoea hypopnea index > 19^ or, if not available, self-reported doctor-diagnosed	8.1
Hearing impairment	Audiometry best ear 4-frequency average > 25 dB* or, if not available*,* hearing impairment affecting daily life frequently/constantly	7.1
Diabetes	Self-reported doctor-diagnosed diabetes (not only during pregnancy) or fasting blood glucose > 7.0 mmol/L or glycosylated haemaglobin (HbA1c) > 6.5%	7
Cancer (non-skin cancer)	Self-reported doctor-diagnosed	6.7
COPD	Post-bronchodilator FEV1/FVC < 0.7 and FEV1% predicted < 80% or, if spirometry not available, self-reported doctor-diagnosed COPD	5.7
Kidney disease	Self-reported doctor-diagnosed kidney disease or estimated glomerular filtration rate < 60 ml/min	5.2
Osteoporosis	DXA measured femur or AP spine T-score ≤ − 2.5^^ or, if not available*,* self-reported doctor-diagnosed	3.4
Stroke	Self-reported doctor-diagnosed stroke, TIA *or* carotid surgery (endarterectomy or stent)	2.1
Peripheral artery disease	Ankle brachial index < 0.9 for right or left side^^^	1.9

Measured using ^Apnealink™ (Resmed), ^^Lunar Prodigy Pro Densitometer (GE), ^^^Vascular profiler VP1000™ (Omran), * Audiometry measured hearing loss of at least 25 dB (dB) (mean of thresholds at 500, 1000, 2000 and 4000 Hz)

Abbreviations:” ALT = Alanine transferase; PHQ9 = Patient Health Questionnaire-9; GAD7 = Generalised anxiety disorder – 7 item scale; COPD = Chronic obstructive pulmonary Disease; FEV1 = Forced expired volume in 1 s; FVC = Forced vital capacity: DXA = Dual energy x-ray absorptiometry; AP = Anterior-posterior; TIA = transient ischemic attack

### Prevalence estimates and patterns of multimorbidity

The observed prevalence of each possible pair and triplet combinations of concurrent conditions was determined in the BHAS cohort. Pairs and triplets of conditions that occurred more often than expected by chance (determined by a chi-square test for statistical independence between conditions) were identified and the observed/expected (O/E) prevalence ratio presented [18, 35]. The expected chance prevalence under independence is calculated as the product of the observed prevalences of each condition in the pair or triplet.

Latent Class Analysis (LCA) was used to identify groups (latent classes) of individuals with similar disease profiles, that is, similar patterns of multi-morbidity. LCA is a model-based statistical technique that is increasingly recommended and used for this purpose [18, 21, 36]. In our study the four class model was judged optimal and we present the proportion of the population that is estimated to be in each class, the estimated probabilities that a person in each class has each of the 21 conditions and the mean number of conditions for each class. The multimorbidity profile of each class was used to interpret and name the classes. Further details of the LCA analysis are provided in Supplementary File 2.

Multivariable logistic and linear regression modelling were used to identify the participant characteristics that were related to latent class membership and overall number of conditions respectively. Regression results are presented as estimates with *p*-values. Statistical analyses were performed using SAS® version 9.3 (and including the Proc LCA extension).

## Results

Complete data on 21 chronic conditions were available for 5029 participants. The average age of the participants was 54 years (range 45–69 years); 55% were female and 98% were Caucasian. Demographic details of the cohort are provided in Supplementary Table S1. Comparison with the Australian National Health Survey 2011 for common risk factors for disease showed that the cohort was similar to the Australian population on a number of key health indicators including prevalence of obesity, hypertension, hypercholesterolemia, and physical activity rates. The prevalence of current tobacco smoking was 10% in the BHAS cohort, lower than the national average for this age group (15%).

### Prevalence of individual conditions and number of conditions per person

The prevalence and definitions of individual chronic conditions are shown in Table 1. The number of co-occurring conditions ranged from 0 to 13 (mean 2.75, SD = 1.84), with 73% of participants having 2 or more conditions, 44% of participants having one or two conditions and 49% having three or more conditions. Only 7% of the cohort had no conditions. The mean and number of conditions increased with age, lower income and lower education level in both men and women (Supplementary Table S1).

### Relationship between number of conditions and participant characteristics

Multiple linear regression modelling showed that female gender; older age; increased waist circumference; tobacco smoking; reduced physical activity; and family history of asthma, diabetes, myocardial infarct and cancer were independently associated with increased number of chronic conditions (Supplementary Table S2). When standard drinks of alcohol per week and the dietary variables of meat meals per week, fish meals per week, vegetable serves per day, fruit serves per day, were added to the model they did not reach statistical significance (*p* = 0.28 for alcohol and *p* > 0.05 for dietary variables) and so were not included in the final presented model.

### Common pairs of conditions

There was a total of (21 × 20)/2 = 210 possible pairs of conditions and all were observed with a prevalence that varied from 0.02 to 13.42% (Table 2). There were 13 pairs of conditions with observed prevalence > 1.5% and an O/E ratio > 1.5 (*p* < 0.0001 for all) (Table 2). Arthritis *and* bowel disease was the most prevalent pair. Arthritis, low back pain and depression–anxiety each featured in at least three of these 13 pairs of conditions.

**Table 2:** All pairs of conditions with prevalence > 1.5% and observed/expected ratio > 1.5 (and *p* < 0.0001)

Co-occurring pairs of conditions			Prevalence (%)	O/E
Asthma	+	COPD	2.41	2.95
Depression–anxiety	+	Low back pain	3.86	2.21
Arthritis	+	Low back pain	4.71	2.2
Bronchitis	+	COPD	1.89	1.86
Low back pain	+	Bronchitis	2.66	1.78
Diabetes	+	Eye condition	1.73	1.7
Diabetes	+	Liver disease	2.66	1.66
Depression–anxiety	+	OSA	2.64	1.6
Thyroid disease	+	Arthritis	3.46	1.59
Kidney disease	+	Depression anxiety	1.67	1.57
Liver disease	+	OSA	2.86	1.54
Bowel disease	+	Low back pain	3.02	1.53
Bowel disease	+	Arthritis	8.81	1.51

Note. Total prevalence of co-occurring conditions was 73%

### Common triplets of conditions

There was a total of (21 × 20 × 19)/6 = 1330 possible triplets of conditions and 1261 were observed with prevalence from 0.02 to 4.97%. There were 27 triplets with a prevalence > 1.5% and O/E ratio > 1.5 (p < 0.0001 for all) (Supplementary Table S3). Bowel disease, arthritis and atopy was the most prevalent triplet. Arthritis featured in 16 of these 27 triplets of conditions and bowel disease and depression–anxiety each featured in 11.

### Patterns of multimorbidity – latent class analysis (LCA)

The morbidity patterns for the 4-class model are shown in Table 3. The classes were defined as follows: **Class 1** (70% of participants) “healthy” – below average prevalence for 20/21 conditions and average of 1.95 conditions; **Class 2** (11%) “respiratory and atopy” – well above average prevalence of asthma, COPD and atopy and below average prevalence of most other conditions, with an overall average of 3.65 conditions; **Class 3** (14%) “multimorbid non-cardiometabolic”– well above average prevalence of thyroid disease, bowel disease, osteoporosis, arthritis, low back pain, asthma, COPD, peripheral arterial disease, hearing impairment, bronchitis and eye conditions, with an overall average of 4.17 conditions; **Class 4** (5%) “multimorbid cardio-metabolic and other”- well above average prevalence of heart disease, stroke, peripheral arterial disease, diabetes, kidney disease, liver disease, bowel disease, depression–anxiety, arthritis, low back pain, sleep apnoea, asthma, bronchitis, chronic hearing impairment and eye conditions, with an overall average of 6.32 conditions.

**Table 3:** Morbidity characteristics of individuals assigned to Classes of multimorbidity

	ALL	CLASS 1	CLASS 2	CLASS 3	CLASS 4
	(N = 5029)	Relatively healthy	Predominant respiratory & atopy	Multi-morbid non-cardio-metabolic	Multi-morbid cardio-metabolic and other
Number assigned to class	**5029 (100%)**	**3541 (70.4%)**	**529 (10.5%)**	**691 (13.7%)**	**268 (5.3%)**
Mean number of conditions	**2.75**	**1.95**	**3.65**	**4.77**	**6.32**
Condition	**Prevalence** **(%)**	**Probability** **(%)**	**Probability** **(%)**	**Probability** **(%)**	**Probability** **(%)**
Atopy	54.4	52.3	82.0	40.8	62.7
Arthritis	25.1	13.3	24.6	77.1	48.1
Bowel disease	23.3	16.5	21.9	54.1	35.4
Liver disease	23.1	21.9	23.3	15.8	57.5
Depression/anxiety	20.5	13.3	14.7	54.6	38.4
Bronchitis	17.6	11.8	20.6	37.8	36.9
Eye condition	14.6	10.8	11.9	30.4	29.1
Asthma	14.2	0.1	99.1	14.5	32.5
Heart disease	9.7	7.7	9.1	7.4	44.0
Thyroid disease	8.6	5.2	7.6	25.8	12.3
Low back pain	8.5	1.6	4.7	37.5	32.5
Skin cancer	8.2	8.7	4.7	8.7	6.3
OSA	8.1	5.8	2.5	12.4	37.3
Hearing impairment	7.1	6.0	3.6	11.6	16.4
Diabetes	7.0	4.2	0.0	4.3	64.6
Non skin cancer	6.7	5.0	6.2	14.2	10.8
COPD	5.7	2.7	19.3	8.4	13.1
Kidney disease	5.2	3.4	2.6	10.0	20.9
Osteoporosis	3.4	2.7	4.2	7.7	0.4
Stroke	2.1	0.9	0.8	2.5	20.1
Peripheral artery disease	1.9	1.2	1.5	1.6	12.3

Arthritis, low back pain, bowel disease, depression–anxiety and bronchitis feature prominently in common pairs and triplets of conditions that occur much more often than expected by chance (all triplets O/E > 3.9) and all of these conditions have high prevalence in multi-morbid classes 3 and 4.

Asthma, COPD and atopy is a common triplet that also occurs much more often than expected by chance (O/E = 3.95) and these three conditions have high prevalence in “respiratory and atopy” class 2.

### Characteristics of multimorbidity

#### Morbidity patterns versus number of conditions

There was a very strong association between having 0–2 conditions and being in ‘healthy’ class 1 (Supplementary Table S4). Of the people with 0–2 conditions, 95% were assigned to ‘healthy’ class 1 and 69% of people in class 1 had 0–2 conditions. Therefore, correlates and predictors of being in ‘healthy’ class 1 and having 0–2 conditions are essentially the same. People with 3–6 conditions make up 46% of the total sample and were distributed across all four classes. Although there was a strong association between having 7+ conditions and being in unhealthy class 3 or 4 (94% of people with 7–13 conditions were assigned to unhealthy classes 3 and 4), the morbidity pattern was different for class 3 and class 4. Thus, amongst people with three or more conditions, the number of conditions alone was not sufficient to discern the pattern of multi-morbidity and correlates. Therefore predictors of being in class 3 and 4 may be quite different and will not necessarily be the same as correlates of the overall number of conditions.

#### Characteristics across assigned multimorbidity classes

Participants in the multi-morbid classes 3 and 4 were, on average, 3 years older than those in classes 1 and 2 (Table 4). Classes 1 and 2 had approximately equal numbers of men and women whereas multi-morbid class 3 had a considerable majority of females and multi-morbid class 4 a considerable majority of males. All characteristics were significantly different across classes (all *p* < 0.001).

**Table 4:** Comparison of participant characteristics across assigned multimorbidity classes

	ALL	Class 1	Class 2	Class 3	Class 4	
		Relatively healthy	Predominantly respiratory & atopy	Multi-morbid non-cardiometabolic	Multi-morbid cardiometabolic and other	
Characteristic	(n = 5029)	(***n*** = 3541)	(***n*** = 529)	(***n*** = 691)	(***n*** = 268)	P - value*
Age (mean years)	58.0	57.5	57.1	60.2	61.0	< 0.001
Sex (Female, %)	54.8	52	61	73	36	< 0.001
Waist circumference (mean cm)	92.2	91.0	90.8	93.3	108.6	< 0.001
Tobacco smoking:						
Never *(%)*	47	48	49	43	35	
Former *(%)*	43	43	43	41	51	
Current < 15 cigarettes per day (%)	4	4	3	7	4	< 0.001
Current 15 cigarettes per day *(%)*	6	5	5	9	9	
Moderate/vigorous physical activity (mean total hours per week)	9.5	10.1	9.0	7.8	7.4	< 0.001
Alcohol consumption (mean total glasses per week)	11.5	11.9	10.9	9.7	12.1	0.001
First degree relatives^#^ with:						
Asthma	0.34	0.29	0.59	0.37	0.39	< 0.001
Diabetes	0.37	0.34	0.36	0.42	0.61	< 0.001
Myocardial infarct	0.5	0.47	0.44	0.62	0.69	< 0.001
Cancer *(any type)*	0.86	0.82	0.84	1.03	0.91	< 0.001

* P-value from chi-square test for categorical characteristic or from ANOVA for quantitative characteristic

# Condition reported in biological mother, father, brother(s), sister(s) – mean number

#### Relationship between membership of particular multimorbidity class and participant characteristics

Multiple logistic regression of assigned membership of each class (vs class 1) identified that individuals were more likely to be classified as “respiratory and atopy” (class 2) than ‘healthy’ (class 1) if they were female and had family history of asthma (Table 5).

**Table 5:** Relationship between membership of particular multi-morbidity class and participant characteristics

Comparison	Class 2 vs Class 1		Class 3 vs Class 1		Class 4 vs Class 1	
Characteristic	Estimated odds ratio	P Value	Estimated odds ratio	P Value	Estimated odds ratio	P Value
Sex (female vs male)	1.409	0.0023	3.227	< 0.0001	1.076	0.6511
Age (per year)	0.996	0.6518	1.089	< 0.0001	1.113	< 0.0001
Waist circumference (per cm)	1.006	0.1275	1.026	< 0.0001	1.097	< 0.0001
Tobacco smoking						
Current 15 cigs/day vs never	0.982	0.9370	3.382	< 0.0001	3.216	< 0.0001
Current < 15 cigs/day vs never	0.816	0.4383	2.527	< 0.0001	1.915	0.0737
Former smoker vs Never smoked	0.959	0.6769	1.084	0.3951	1.228	0.1869
Weekly moderate/vigorous physical activity (per hr)	0.995	0.2739	0.993	0.0703	0.986	0.0251
First degree relative with:						
Asthma	1.834	<.0001	1.189	0.0139	1.335	0.0080
Diabetes	0.981	0.8029	1.076	0.2792	1.437	0.0001
Myocardial infarct	0.882	0955	1.141	0.0293	1.291	0.0065
Cancer (any)	0.999	0.9790	1.237	< 0.0001	1.061	0.4747

Individuals were more likely to be classified as “multi-morbid non-cardio-metabolic” (class 3) than ‘healthy’ (class 1) if they were female, older, had a larger waist circumference, smoked, and had a family history of asthma, myocardial infarction or cancer.

Individuals were more likely to be classified as “multi-morbid cardio-metabolic and other” (class 4) than ‘healthy’ (class 1) if they were older, had a larger waist circumference, smoked, did less physical activity, and had a family history of asthma, diabetes and myocardial infarction.

These results confirm that predictors of being in classes 2, 3 and 4 differ and are not the same as predictors of the number of conditions.

## Discussion

In this study, we have used an extensive range of clinically measured and self-reported chronic conditions and two complementary analytical approaches to describe the prevalence and patterns of multimorbidity in a community cohort of middle-aged adults. Our first approach, using single disease counts of pairs and triplets of conditions, identified that multimorbidity is prevalent and that some combinations of conditions occur more frequently than expected by chance. Our second approach, using LCA, identified four unbiased classes of multimorbidity, with differences in membership of each class characterised by both higher probability of the number of chronic conditions and a clinically-distinct groupings of conditions. The predictors of chronic disease load (number of conditions) differed from those of patterns of multimorbidity.

Using single disease counts, we showed that, although the majority (73%) of adults in this cohort had two or more chronic conditions, the overall prevalence of specific combinations of disease was relatively low. The most prevalent pair (atopy and arthritis) had a prevalence of just over 13% and the most prevalent triplet (arthritis, bowel disease and atopy) was observed in under 5% of the cohort. By calculating O/E prevalence ratios, we were able to identify combinations of disease that occurred more frequently than expected by chance. Some of the combinations identified in this cohort have obvious pathological links or are well known to co-exist clinically. Asthma and COPD either as a pair, or along with atopy in a triplet, were combinations with high O/E ratios and point towards well-documented inflammatory links between allergy and respiratory function. Although we mainly used spirometry to define COPD, the appearance of this condition alongside asthma may point towards overlap of symptoms or diagnostic mislabelling and highlights the need for further research into how these conditions might be better characterised [37].

Arthritis, bowel disease and low back pain were the most common conditions appearing in highest ranked O/E combinations alongside depression-anxiety. Arthritis featured in 60% of the highest-ranking triplets of conditions and bowel disease and depression-anxiety featured in 40%. Depression-anxiety featured in over one-third of all triplets identified and was highly prevalent among LCA Class 4 with the highest condition load. Depression is a common feature of multimorbidity [38] and has been associated with a range of chronic conditions including cardiac disease, stroke, loss of hearing, loss of vision, and chronic lung diseases such as asthma and COPD [39, 40]. In our study, depression-anxiety often featured in combination with arthritis, back pain and bowel disease. Previous studies have shown high prevalence of depression and anxiety in inflammatory bowel disorders such as ulcerative colitis and Crohn’s disease [41, 42]. Depression is also a common comorbid feature in different arthritis subtypes [43] and can worsen both the severity of and disability caused by chronic low back pain [44]. As depression is a treatable illness, a rational approach may be to screen for and treat comorbid depression in patients presenting with these patterns of multimorbidity.

Other combinations of disease identified in our study may point towards shared aetiologies. The high co-occurrence of arthritis with bowel disease has been described in previous studies and was a common combination identified in both our disease counts (7/27 triplets) and occurred in high prevalence in LCA Classes 3 and 4 [45, 46]. Previous studies have suggested changes in gut bacteria as potentially implicated in the development of joint inflammation, or alternatively recruitment of gut lymphocytes or activated macrophages in joint inflammation [47].

Common risk factors such as obesity, tobacco smoking, physical inactivity, and family history may underlie some of these patterns, yet predictors of latent class membership showed distinct differences. Our analysis of the lifestyle factors underlying class membership or the number of chronic conditions did not find alcohol consumption or diet variables (data not shown) to be significant variables.

### LCA patterns

The largest disease burden was evident in Classes 3 and 4, which together comprised around 20% of the cohort. Individuals assigned to Class 3 *multi-morbid non-cardiometabolic* had an average of over 4 chronic conditions and showed a higher probability of musculoskeletal conditions (arthritis, low back pain and osteoporosis); conditions resulting in sensory deficits (hearing impairment and eye conditions); and non-cardiovascular conditions including bowel, thyroid and kidney diseases. Individuals in this category were more likely to be female (73%) and older than those in classes 1 and 2. While only 5% of the cohort was assigned to Class 4, this class was 64% male and had the highest disease load with an average of six co-occurring chronic conditions. Cardiometabolic conditions including heart disease, diabetes, stroke, and peripheral artery disease along with liver disease and sleep apnoea were highly prevalent in this group. Individuals in Class 4 were much more likely to be male, older and obese.

Similar to the findings for common pairs and triplets, Class 2 was characterised by above-average prevalence of respiratory and allergy conditions but did not significantly differ from Class 1 (relatively healthy) in terms of age, obesity or smoking status. This is interesting because this class shows a distinct clustering of multimorbidity that was not associated with increased age (the average age was lower in this group compared with Classes 3 and 4) or with behavioural risk factors related to poor lung function, such as smoking and excess abdominal fat (compared with Class 1). Family history of asthma was a significant predictor of membership of this group, perhaps highlighting a strong genetic and early life component underlying this pattern of multimorbidity.

Comparing our findings with previous studies that identify multimorbidity patterns is difficult due to the heterogeneity in the populations and age-ranges sampled, disease type and number of conditions included, as well as variation in data sources and analytical methods. Nonetheless a systematic review of 14 studies on non-random patterns of multimorbidity provides support for three broad categories consisting of cardiovascular and metabolic diseases, musculoskeletal disorders and mental health disorders [27]. Using disease counts, similar patterns have been described in Australian samples. Britt et al. found that arthritis, chronic back pain and depression-anxiety conditions were the most prevalent combinations of conditions, along with vascular disease, in older primary-care patients [20]. Holden et al. used exploratory factor analysis to describe patterns and identified a dominant group with arthritis and irritable bowel disease [17].

Relatively few published studies have employed LCA to describe multimorbidity classes in general population samples. An Australian study of over 4500 adults with mean age of 69 years included 11 self-reported disease conditions resulting in four multimorbidity LCA classes [18]. Similar to our findings, the majority of the study sample (> 55%) were assigned to a minimally diseased (‘healthy’) group. The remainder were assigned to groups characterised by dominant presence of either: arthritis *and* asthma *and* depression–anxiety; high blood pressure *and* diabetes, or; cancer *and* heart disease *and* stroke. The clustering of conditions in that study was largely consistent across the multiple analytical techniques employed, which included pair/triplet disease combinations and cluster analyses. While we identified broadly similar combinations of conditions and clusters in our study sample, it is likely that inclusion of more conditions in our analyses resulted in a distinct grouping of cardio-metabolic conditions that included diabetes, heart disease and stroke into a single class (Class 4). A study using 15 self-reported conditions in over 160,000 Danish citizens with mean age 47 years, identified seven classes of multimorbidity, three of which were similar to those identified in our analyses [21]. These include a distinct “musculoskeletal class” characterised by high probability of arthritis, osteoporosis and back injuries; a “complex cardio-metabolic disorders” class with high probability of diabetes, heart disease and stroke, similar to our Class 4; and a third class termed “asthma–allergy”, similar to our Class 2 but which did not include COPD.

A US study of over 14,000 adults aged over 65 years used 13 self-reported conditions identified six latent multimorbidity classes [36]. Only one-third were assigned to a “Minimal disease” group and the remainder spread between groups characterised by vascular, non-vascular, cardio-stroke-cancer or neurological disease, and a ‘Very sick’ group with above average prevalence of all conditions. These findings differ from our study in that we found distinct differences between predictors of class membership and number of diseases, and that the majority of our sample (70%) fell within a relatively healthy (minimal disease) group, the latter of which may be explained by the overall younger age of our cohort.

Comparing the patterns of multimorbidity identified in our study and others conducted in high income countries, with patterns identified in low-middle income countries is difficult given the different nature of disease burden and the limited availability of studies. Nonetheless a recent LCA analysis in cohorts from both low and high income countries identified three classes, two of which labelled “cardio-metabolic” and “healthy” were similar to those found in our study and others [27, 48]. However the proportion of individuals in each of the multimorbidity classes differed across regions highlighting the difficulties in comparing multimorbidity burden and patterns across different socio-economic and epidemiological settings.

### Strengths

The strengths of our study include a high participation and completion rate in a moderately large community-dwelling cohort, assessed at an age where chronic disease load often begins to manifest. By using a large number of conditions (over 50% of which were defined by physical or biochemical clinical measure or validated instrument score) and including those conditions that are current and chronic (long-lasting symptoms and likely to impact on daily activities) we have captured a comprehensive range of conditions of ageing that have not been able to be included in previous studies of multimorbidity.

### Limitations

Our sample was drawn from a regional urban community; however the cohort was similar on a number of key health indicators to the general Australian population. Current medication use, although available, was not included in the definition or criteria for any of the conditions so as to avoid potential misclassification errors that may arise from off-label prescribing or under-reporting. Without an objective clinical marker for arthritis (similar to other studies), we have relied on self-reported doctor diagnosis, but we used validated instrument scores to capture musculoskeletal conditions including low back pain and a wide range of gastrointestinal disorders in our definition of bowel disease, some of which have not often been included in previous studies of multimorbidity patterns [32].

When defining multimorbidity, “health conditions” may include diseases, risk factors or symptoms. The present analysis has included predominantly conditions that are diseases or symptoms. We therefore did not include conditions such as obesity or hypertension. While hypertension can be considered a cardiovascular condition, it was not included in this analysis because of its very strong risk factor association with stroke, heart disease, kidney disease and diabetes and is thus a somewhat redundant aspect of the cardio-metabolic profile and patterns identified. Decisions on what to include when defining multimorbidity depend on the purpose of the definition, e.g. assessing effects on perceived future risk, or effects on current function and disability, or effects on health care costs [48]. The present study aimed at examining how diseases and symptoms co-occurred and might be predicted.

## Conclusion

Our findings indicate that multimorbidity is common among middle-aged adults and some conditions co-occur in clinically distinct patterns and at higher prevalence than expected by chance. The identification of non-random patterns of multimorbidity may guide further mechanistic studies into shared biological and environmental causes of co-occurring conditions of ageing. As the Busselton Healthy Ageing Study is designed as a longitudinal survey, the conduct of future phases and analyses will track and relate patterns of multimorbidity to prevalent and incident cognitive and physical disability and use of healthcare resources. The findings of these studies will be of major importance to the primary rather than specialist care management of older individuals in whom multiple physical and mental health conditions require a holistic view.

## Supplementary Information

**Additional file 1.** Supplementary File 1. BHAS Phase 1 Questionnaire 2010–2015.

**Additional file 2.** Supplementary File 2. Statistical Supplement. Latent Class Analysis method.

**Additional file 3.** Supplementary Table S1. Mean, median and maximum number of conditions by gender and demographic groups.

**Additional file 4.** Supplementary Table S2. Relationship between number of conditions and participant characteristics.

**Additional file 5 **Supplementary Table S3. All triplets of conditions with prevalence > 1.5% and observed/expected (O/E) ratio > 1.5 (and *p* value< 0.0001).

**Additional file 6.** Supplementary Table S4. Morbidity patterns and number of conditions. Number of participants with each number of conditions and percentage within each morbidity pattern.

## Data Availability

Data is available by application to the Busselton Population Medical Research Institute.

## Abbreviations

AP: Anterior-posterior
ABI: Ankle-Brachial Index
AIC: Akaike Information Criterion
ALT: Alanine transferase
ANOVA: Analysis of variance
BHAS: Busselton Healthy Ageing Study
BIC: Bayesian Information Criterion
cm: centimetre
COPD: Chronic obstructive pulmonary disease
dB: decibels
DXA: Dual energy x-ray absorptiometry
ECG: electrocardiogram
FEV1: Forced expired volume in 1 s
FVC: Forced vital capacity
GAD7: Generalised anxiety disorder – 7 item scale
HbA1c: glycated haemoglobin
Hz: Hertz
LCA: Latent Class Analysis
ml/min: millilitres per minute
mm: millimetre
mmol/L: millimoles per litre
O/E: Observed/expected
PHQ9: Patient Health Questionnaire-9
SD: Standard deviation
TIA: Transient ischemic attack
U/L: Units per litre

## References

[1] Johnston MC, Crilly M, Black C, Prescott GJ, Mercer SW (2019). Defining and measuring multimorbidity: a systematic review of systematic reviews. Eur J Pub Health.

[2] Chandraratne NK, Pathirathna K, Harrison C, Siriwardena AN (2018). A comparison of policies and guidelines related to multimorbidity in the UK, Australia and Sri Lanka. Aust J Gen Pract.

[3] Nunes BP, Flores TR, Mielke GI, Thume E, Facchini LA. Multimorbidity and mortality in older adults: a systematic review and meta-analysis. Arch Gerontol Geriatr. 2016;67:130–8. 10.1016/j.archger.2016.07.008. 10.1016/j.archger.2016.07.008 27500661

[4] Fortin M, Bravo G, Hudon C, Lapointe L, Almirall J, Dubois MF, Vanasse A (2006). Relationship between multimorbidity and health-related quality of life of patients in primary care. Qual Life Res.

[5] Barnett K, Mercer SW, Norbury M, Watt G, Wyke S, Guthrie B (2012). Epidemiology of multimorbidity and implications for health care, research, and medical education: a cross-sectional study. Lancet..

[6] Fortin M, Bravo G, Hudon C, Lapointe L, Dubois MF, Almirall J (2006). Psychological distress and multimorbidity in primary care. Ann Fam Med.

[7] Fabbri E, An Y, Zoli M, Tanaka T, Simonsick EM, Kitner-Triolo MH, Studenski SA, Resnick SM, Ferrucci L (2016). Association between accelerated multimorbidity and age-related cognitive decline in older Baltimore longitudinal study of aging participants without dementia. J Am Geriatr Soc.

[8] Sheridan PE, Mair CA, Quinones AR (2019). Associations between prevalent multimorbidity combinations and prospective disability and self-rated health among older adults in Europe. BMC Geriatr.

[9] Vogel I, Miksch A, Goetz K, Ose D, Szecsenyi J, Freund T (2012). The impact of perceived social support and sense of coherence on health-related quality of life in multimorbid primary care patients. Chronic Illn.

[10] Troelstra SA, Straker L, Harris M, Brown S, van der Beek AJ, Coenen P (2020). Multimorbidity is common among young workers and related to increased work absenteeism and presenteeism: results from the population-based Raine study cohort. Scand J Work Environ Health.

[11] Abebe F, Schneider M, Asrat B, Ambaw F (2020). Multimorbidity of chronic non-communicable diseases in low- and middle-income countries: A scoping review. J Comorb.

[12] Muth C, Blom JW, Smith SM, Johnell K, Gonzalez-Gonzalez AI, Nguyen TS, Brueckle MS, Cesari M, Tinetti ME, Valderas JM (2019). Evidence supporting the best clinical management of patients with multimorbidity and polypharmacy: a systematic guideline review and expert consensus. J Intern Med.

[13] Kernick D, Chew-Graham CA, O'Flynn N (2017). Clinical assessment and management of multimorbidity: NICE guideline. Br J Gen Pract.

[14] National Institute for Health and Care Excellence. Multimorbidity: clinical assessment and management. NICE guideline [NG56]. 2016. https://www.nice.org.uk/guidance/ng56. Accessed 10 July 2020.

[15] Nadeeka KC, Pathirathna K, Christopher H, Siriwardena AN (2018). A comparison of policies and guidelines. Austr J Gen Pract.

[16] Fortin M, Stewart M, Poitras ME, Almirall J, Maddocks H (2012). A systematic review of prevalence studies on multimorbidity: toward a more uniform methodology. Ann Fam Med.

[17] Holden L, Scuffham PA, Hilton MF, Muspratt A, Ng SK, Whiteford HA (2011). Patterns of multimorbidity in working Australians. Popul Health Metrics.

[18] Islam MM, Valderas JM, Yen L, Dawda P, Jowsey T, McRae IS (2014). Multimorbidity and comorbidity of chronic diseases among the senior Australians: prevalence and patterns. PLoS One.

[19] Walker AE (2007). Multiple chronic diseases and quality of life: patterns emerging from a large national sample, Australia. Chronic Illn.

[20] Britt HC, Harrison CM, Miller GC, Knox SA (2008). Prevalence and patterns of multimorbidity in Australia. Med J Aust.

[21] Larsen FB, Pedersen MH, Friis K, Glümer C, Lasgaard M (2017). A Latent Class Analysis of Multimorbidity and the Relationship to Socio-Demographic Factors and Health-Related Quality of Life. A National Population-Based Study of 162,283 Danish Adults. PLoS One.

[22] Diederichs C, Berger K, Bartels DB (2011). The measurement of multiple chronic diseases--a systematic review on existing multimorbidity indices. J Gerontol A Biol Sci Med Sci.

[23] Huntley AL, Johnson R, Purdy S, Valderas JM, Salisbury C (2012). Measures of multimorbidity and morbidity burden for use in primary care and community settings: a systematic review and guide. Ann Fam Med.

[24] Nicholson K, Almirall J, Fortin M (2019). The measurement of multimorbidity. Health Psychol.

[25] Fortin M, Haggerty J, Sanche S, Almirall J (2017). Self-reported versus health administrative data: implications for assessing chronic illness burden in populations. A cross-sectional study. CMAJ Open.

[26] Lujic S, Simpson JM, Zwar N, Hosseinzadeh H, Jorm L (2017). Multimorbidity in Australia: comparing estimates derived using administrative data sources and survey data. PLoS One.

[27] Prados-Torres A, Calderón-Larrañaga A, Hancco-Saavedra J, Poblador-Plou B, van den Akker M. Multimorbidity patterns: a systematic review. J Clin Epidemiol. 2014;67(3):254–66. 10.1016/j.jclinepi.2013.09.021. 10.1016/j.jclinepi.2013.09.021 24472295

[28] Bastian LA, Brandt CA, Justice AC (2017). Measuring multimorbidity: a risky business. J Gen Intern Med.

[29] James A, Hunter M, Straker L, Beilby J, Bucks R, Davis T (2013). Rationale, design and methods for a community-based study of clustering and cumulative effects of chronic disease processes and their effects on ageing: the Busselton healthy ageing study. BMC Public Health.

[30] Levey AS, Stevens LA, Schmid CH, Zhang YL, Castro AF, Feldman HI (2009). A new equation to estimate glomerular filtration rate. Ann Intern Med.

[31] Craig CL, Marshall AL, Sjostrom M, Bauman AE, Booth ML, Ainsworth BE (2003). International physical activity questionnaire: 12-country reliability and validity. Med Sci Sports Exerc.

[32] Fairbank JC, Pynsent PB (2000). The Oswestry disability index. Spine (Phila Pa 1976).

[33] Kroenke K, Spitzer RL, Williams JB (2001). The PHQ-9: validity of a brief depression severity measure. J Gen Intern Med.

[34] Spitzer RL, Kroenke K, Williams JB, Lowe B (2006). A brief measure for assessing generalized anxiety disorder: the GAD-7. Arch Intern Med.

[35] Harrison C, Henderson J, Miller G, Britt H (2016). The prevalence of complex multimorbidity in Australia. Aust N Z J Public Health.

[36] Whitson HE, Johnson KS, Sloane R, Cigolle CT, Pieper CF, Landerman L, Hastings SN (2016). Identifying patterns of multimorbidity in older Americans: application of latent class analysis. J Am Geriatr Soc.

[37] James AL, Knuiman MW, Divitini ML, Hui J, Hunter M, Palmer LJ, Maier G, Musk AW (2010). Changes in the prevalence of asthma in adults since 1966: the Busselton health study. Eur Respir J.

[38] Read JR, Sharpe L, Modini M, Dear BF. Multimorbidity and depression: a systematic review and meta-analysis. J Affect Disord. 2017;221:36–46. 10.1016/j.jad.2017.06.009. 10.1016/j.jad.2017.06.009 28628766

[39] Stanton R, Rosenbaum S, Rebar A, Happell B (2019). Prevalence of chronic health conditions in Australian adults with depression and/or anxiety. Iss Ment Health Nurs.

[40] Huang CQ, Dong BR, Lu ZC, Yue JR, Liu QX. Chronic diseases and risk for depression in old age: a meta-analysis of published literature. Ageing Res Rev. 2010;9(2):131–41. 10.1016/j.arr.2009.05.005. 10.1016/j.arr.2009.05.005 19524072

[41] Mikocka-Walus A, Knowles SR, Keefer L, Graff L (2016). Controversies revisited: a systematic review of the comorbidity of depression and anxiety with inflammatory bowel diseases. Inflamm Bowel Dis.

[42] Neuendorf R, Harding A, Stello N, Hanes D, Wahbeh H. Depression and anxiety in patients with inflammatory bowel disease: a systematic review. J Psychosom Res. 2016;87:70–80. 10.1016/j.jpsychores.2016.06.001. 10.1016/j.jpsychores.2016.06.001 27411754

[43] Brooks JM, Titus AJ, Polenick CA, Orzechowski NM, Reid MC, MacKenzie TA (2018). Prevalence rates of arthritis among US older adults with varying degrees of depression: findings from the 2011 to 2014 National Health and nutrition examination survey. Int J Geriatr Psychiatry.

[44] Carley JA, Karp JF, Gentili A, Marcum ZA, Reid MC, Rodriguez E, Rossi MI, Shega J, Thielke S, Weiner DK (2015). Deconstructing chronic low Back pain in the older adult: step by step evidence and expert-based recommendations for evaluation and treatment: part IV: depression. Pain Med.

[45] Karreman MC, Luime JJ, Hazes JMW, Weel A (2017). The prevalence and incidence of axial and peripheral Spondyloarthritis in inflammatory bowel disease: a systematic review and Meta-analysis. J Crohns Colitis.

[46] Brakenhoff LK, van der Heijde DM, Hommes DW, Huizinga TW, Fidder HH. The joint-gut axis in inflammatory bowel diseases. J Crohns Colitis. 2010;4(3):257–68. 10.1016/j.crohns.2009.11.005. 10.1016/j.crohns.2009.11.005 21122514

[47] Speca S, Dubuquoy L (2017). Chronic bowel inflammation and inflammatory joint disease: pathophysiology. Joint Bone Spine.

[48] Willadsen TG, Bebe A, Koster-Rasmussen R, Jarbol DE, Guassora AD, Waldorff FB (2016). The role of diseases, risk factors and symptoms in the definition of multimorbidity - a systematic review. Scand J Prim Health Care.

