# Supplementary material for: Prevalence and patterns of multimorbidity in Australian baby boomers: the Busselton healthy ageing study
Source: BMC Public Health. 2021 Aug 11;21:1539. doi: 10.1186/s12889-021-11578-y (PMC8359115; doi:10.1186/s12889-021-11578-y)
Supplement: Supplementary file 1 — Additional file 1. Supplementary File 1. BHAS Phase 1 Questionnaire 2010–2015. [file 12889_2021_11578_MOESM1_ESM.pdf]

Questionnaire ID **2600**

SPECIMEN ID:

FIX LABEL HERE

BUSSELTON ID:

FIX LABEL HERE

The Busselton Health Study

THE UNIVERSITY OF WESTERN AUSTRALIA
Achieving International Excellence

BUSSELTON HEALTHY AGEING STUDY

PARTICIPANT QUESTIONNAIRE

Thank you for taking the time to fill in this important questionnaire.

Please read each question carefully and answer ALL of the questions by following the completion instructions provided below.

All information will remain strictly confidential.

BPMRI

HOW TO COMPLETE THIS FORM

Please use a BLACK pen.

Please shade the circles completely	Please write clearly within the boxes	Please write clearly within the space
●	A B C 1 2 3	PLEASE WRITE IN CAPITAL LETTERS

Please take your time in answering all of the questions.

If you are not sure of an answer, then leave it blank and one of the Study Team will help you with it.

If you make a mistake, or want to change any of your shaded responses, please place a cross through the incorrect response and shade the correct response.

For written responses, please cross out your incorrect response and write your new response just above or below the one you have crossed out.

Questionnaire

By filling in the questionnaire, you are giving your consent for the questionnaire part of this Study. You do not have to answer any questions that you do not wish to. However, the more questions you answer, the better the understanding we will have of the factors that affect your health and well being.

NOTE: We realise that this questionnaire is quite lengthy. You do not have to answer every question, if you do not wish to. However, all information you give us is helpful, so please answer as many questions as you can. Due to the length of the questionnaire, you may wish to complete this document in stages, over a day or so.

If you require further information please contact:
Busselton Health Study
18 West St, Busselton, 6285
(08) 9754 0548

Questionnaire ID **2600**

CONTACT DETAILS

Title:

Surname:

Other name(s):

Maiden name (if applicable):

Date of birth: __ __ / __ __ / __ __ __ __
DD MM YYYY

Postal address:

Suburb Postcode

Residential address (if different from postal address)

Suburb Postcode

Home phone:
Area Code

Work phone:
Area Code

Mobile phone:

Email address (if applicable):

Date you completed this questionnaire: __ __ / __ __ / __ __ __ __
DD MM YYYY

In case we lose contact with you, please nominate someone who would be willing to be contacted to advise us of your current address.

Name:

Home phone:
Area Code

Mobile Phone:

BPMRI

Questionnaire ID 1

DEMOGRAPHY

BUSSELTON ID

FIX LABEL HERE

D1. Date of Birth: __ __ / __ __ / __ __ __ __
DD MM YYYY

D2. Gender: ○ Male ○ Female

D3. Marital status:
○ Single ○ Married ○ Widowed ○ Divorced ○ Separated ○ De Facto

D4. Which of the following describes your current situation?
○ Retired ○ In paid employment or self-employed ○ Unable to work due to sickness or disability
○ Unemployed ○ Doing unpaid or voluntary work ○ Looking after home and/or family
○ None of the above

D5. What has been your usual occupation or job (the one you have worked at the longest)?

D6. For how many years have you worked at this job? __ __ years

D7. What is your current occupation (if different from above)?

D8. What is the average annual total income before tax received by your household?
(please include all wages/salaries, government benefits, pensions, allowances and other income the person usually receives)
○ Less than $20,000 ○ $20,001 to $40,000 ○ $40,001 to $60,000 ○ $60,001 to $80,000
○ $80,001 to $100,000 ○ More than $100,000 ○ None of the above ○ Prefer not to say

D9. What is the highest level of education you have completed?
○ Primary school ○ Secondary school ○ Other educational institute (e.g. TAFE, college)
○ University ○ Did not go to school

D9a. How old were you when you completed your continuous full-time education? __ __ years old

D10. What type of accommodation do you live in?
○ A house ○ Aged care hostel/nursing home
○ A unit/apartment/townhouse ○ Sheltered accommodation (e.g. Aged care village)
○ Mobile or temporary structure (e.g. caravan,park home) ○ None of the above

D11. The dwelling is:
○ Owned outright ○ Owned with a mortgage ○ Being purchased under a rent/buy scheme
○ Being rented ○ Being occupied rent free ○ Being occupied under a life tenure scheme
○ None of the above

D12. For how many years have you lived at this address? __ __ years ○ less than a year

BPMRI

DEMOGRAPHY

D13. Please describe which group(s) best defines the ancestry/ethnicity (based on a mixture of culture, religion, skin colour and language) of you and your biological parents. You may choose more than one group for each person.

A. Caucasian - Australian/NZ (Anglo European), Europe (includes Russia Central and West Asia) & North Mediterranean, America, Canada, South Africa & Zimbabwe.
B. Indigenous Australian - Aboriginal, Torres Stait Islands.
C. Pacific Islander - New Zealand Maori or Pacific Islands, Hawaii, New Guinea.
D. South-East Asia - Brunei, Cambodia, Indonesia, Laos, Malaysia, Myanmar/Burma, Philippines, Singapore, Thailand, Vietnam
E. South Asian - Afghanistan, Bangladesh, Bhutan, India, Maldives, Nepal, Pakistan, Sri Lanka.
F. North East Asia - China, Hong Kong, Japan, Korea, Macau, Taiwan.
G. North Asia - Mongolia, Siberia.
H. Middle Eastern, North Africa, Somalia Peninsular - Algeria, Bahrain, Djibouti, Eritrea, Ethiopia, Egypt, Israel, Iran, Iraq, Jordan, Kuwait, Lebanon, Libya, Oman, Palestinian Territories, Turkey, Turkish Cypriots, QatarSaudi Arabia, Somalia, Syria, Tunisia, United Arab Emirates, Yemen.
I. Sub-Saharan African - Indigenous African, African American.
J. Central/South American - Central/South America.
K. Other
L. Don't Know

	D13a. Your Biological Father	D13b. Your Biological Mother	D13c. You
A. Caucasian	○	○	○
B. Indigenous Australian	○	○	○
C. Pacific Islander	○	○	○
D. South-East Asian	○	○	○
E. South Asian	○	○	○
F. North East Asian	○	○	○
G. North Asian	○	○	○
H. Middle Eastern, North Africa, Somali Peninsular	○	○	○
I. Sub-Saharan African	○	○	○
J. Central/South American	○	○	○
K. Other (please specify below)	○	○	○
L. Don't know	○	○	○

BPMRI

D14. If other, please specify for each:

D14a. Your biological father: ☐☐☐☐☐☐☐☐☐☐☐☐☐☐☐

D14b. Your biological mother: ☐☐☐☐☐☐☐☐☐☐☐☐☐☐☐

D14c. You: ☐☐☐☐☐☐☐☐☐☐☐☐☐☐☐

D15. In which country were you born?

☐☐☐☐☐☐☐☐☐☐☐☐☐☐☐☐☐☐☐☐☐☐

SMOKING HISTORY

It is important that we know if you smoke/have ever smoked, or spend time with people who do.

SM1. Have you ever smoked cigarettes?

○ No ---> please go to SM6

○ Yes *(YES means more than 1 cigarette per day for a year, or 20 packs in a lifetime)*

SM2. Do you currently smoke manufactured or hand-rolled cigarettes? ○ No ○ Yes

SM3. How many cigarettes per day do (did) you smoke? [|] cigarettes per day

SM4. At what age did you start smoking? [|] years old

SM5. How old were you when you last stopped smoking? [|] years old

SM6. How many people in your household currently smoke (excluding yourself)? [|] people

SM7. Are you exposed to tobacco smoke at work? ○ Yes ○ No ○ I don't work

GENERAL HEALTH

Now we would like to learn about your health, including how much physical activity you do.

GH1. In the last <u>2 years</u> have you experienced any of the following? (select all that apply)

○ serious illness, injury or assault to yourself

○ serious illness, injury or assault of a close relative

○ death of a close relative

○ death of a spouse or partner

○ marital separation/divorce

○ financial difficulties

○ None of the above

GH2. Do you have any long-standing illness, disability or infirmity?

○ No ○ Yes

These next questions ask for your views about your health. This information will help keep track of how you feel and how well you are able to do your usual activities. For each of the following questions, please fill in the circle that best describes your answer.

GH3. In general, would you say your health is:

Excellent	Very good	Good	Fair	Poor
○	○	○	○	○

GH4. The following questions are about activities you might do during a typical day. Does your health now limit you in these activites? If so, how much?

	Yes, limited a lot	Yes, limited a little	No, not limited at all
**GH4a. Moderate activities such as moving a table, pushing a vacuum cleaner, bowling or playing golf.**	○	○	○
**GH4b. Climbing several flights of stairs**	○	○	○

BPMRI

GENERAL HEALTH (continued)

GH5. During the past 4 weeks, how much of the time have you had any of the following problems with your work or other regular daily activities as a result of your physical health?

	All of the time	Most of the time	Some of the time	A little of the time	None of the time
**GH5a. Accomplished less than you would like:**	○	○	○	○	○
**GH5b. Were limited in the kind of work or other regular activities:**	○	○	○	○	○

GH6. During the past 4 weeks, how much of the time have you had any of the following problems with your work or other regular daily activities as a result of any emotional problems (such as feeling depressed or anxious)?

	All of the time	Most of the time	Some of the time	A little of the time	None of the time
**GH6a. Accomplished less than you would like:**	○	○	○	○	○
**GH6b. Did work or other activities less carefully than usual:**	○	○	○	○	○

GH7. During the past 4 weeks, how much did pain interfere with your normal work (including both work outside the home and housework)?

Not at all	A little bit	Moderately	Quite a bit	Extremely
○	○	○	○	○

BPMRI

GH8. These questions are about how you feel and how things have been with you during the past 4 weeks. For each question, please give the one answer that comes closest to the way you have been feeling. How much of the time during the past 4 weeks...

	All of the time	Most of the time	Some of the time	A little of the time	None of the time
**GH8a. Have you felt calm and peaceful?**	○	○	○	○	○
**GH8b. Did you have a lot of energy?**	○	○	○	○	○
**GH8c. Have you felt downhearted and depressed?**	○	○	○	○	○

GH9. During the past 4 weeks, how much of the time has your physical health or emotional problems interfered with your social activities (like visiting friends, relatives etc.)?

All of the time	Most of the time	Some of the time	A little of the time	None of the time
○	○	○	○	○

DIET AND NUTRITION

DN1. Approximately how many times each week do you eat the following, including all meals and snacks:
Write '0' if never eaten or eaten less than once a week

[ ][ ] Beef, lamb or pork
[ ][ ] Chicken, turkey or duck
[ ][ ] Fish or seafood
[ ][ ] Cheese
[ ][ ] Processed meat *(include bacon, sausages, salami, devon, burgers, etc.)*

DN2. Approximately how many of the following do you usually eat per week:
Write '0' if never eaten or eaten less than once a week

[ ][ ] Slices or pieces of brown/wholemeal bread each week *(also include multigrain, rye bread, etc.)*
[ ][ ] Bowls of breakfast cereal per week

If you eat breakfast cereal, is it usually:

- ○ bran cereal (allbran, branflakes, etc.)
- ○ biscuit cereal (weetbix, shredded wheat, etc.)
- ○ oat cereal (porridge, etc.)
- ○ muesli
- ○ other (cornflakes, rice bubbles, etc.)

BPMRI

DN3. What type of milk do you usually drink?

○ whole milk ○ reduced fat milk ○ skim milk ○ soy milk ○ other milk ○ I don't drink milk

DN4. Approximately how many serves of vegetables do you usually eat each day? *One serve equals half a cup of cooked vegetables or one cup of salad. Please include potatoes and write '0' if less than one a day.*

[ ][ ] number of serves of COOKED vegetables each day
[ ][ ] number of serves of RAW vegetables each day (e.g. salad)
○ I don't eat vegetables

DN5. Approximately how many serves of fruit or glasses of fruit juice do you usually have each day?
One serve is 1 medium piece or 2 small pieces or 1 cup of diced or canned fruit pieces.

[ ][ ] number of serves of fruit each day
[ ][ ] number of glasses of fruit juice each day
○ I don't eat fruit

DN6. Please fill in the circle if you NEVER eat:

- ○ red meat
- ○ chicken/poultry
- ○ pork/ham
- ○ any meat
- ○ cheese
- ○ cream
- ○ eggs
- ○ dairy products
- ○ fish
- ○ seafood
- ○ wheat products
- ○ sugar

DIET AND NUTRITION (continued)

The following questions are about how often you drink alcohol.

DN7. Over the last 12 months, how often did you drink beer, wine and/or spirits?

	never	less than once a month	1-3 days per month	1 day per week	2 days per week	3 days per week	4 days per week	5 days per week	6 days per week	every day
**DN7a. Beer (low alcohol)**	○	○	○	○	○	○	○	○	○	○
**DN7b. Beer (full strength)**	○	○	○	○	○	○	○	○	○	○
**DN7c. Red wine**	○	○	○	○	○	○	○	○	○	○
**DN7d. White wine (include sparkling wines)**	○	○	○	○	○	○	○	○	○	○
**DN7e. Fortified wines, port, sherry, etc.**	○	○	○	○	○	○	○	○	○	○
**DN7f. Spirits, liqueurs, etc.**	○	○	○	○	○	○	○	○	○	○

BPMRI

IF YOU HAVE NOT CONSUMED ANY ALCOHOL IN THE LAST 12 MONTHS
PLEASE GO TO QUESTION MED1 ON PAGE 7

When answering the next two questions, please convert the amounts you drank into glasses using the examples given below:
For spirits, liqueurs and mixed drinks containing spirits, please count each nip (30ml) as one glass
1 can or stubby of beer = 2 glasses
1 bottle of wine (750ml) = 6 glasses
1 large bottle of beer (750ml) = 4 glasses
1 bottle of port or sherry (750ml) = 12 glasses

DN8. Over the last 12 months, on days when you were drinking,how many glasses of beer, wine and/or spirits altogether did you usually drink?

	1	2	3	4	5	6	7	8	9	10 or more
**Number of glasses per day:**	○	○	○	○	○	○	○	○	○	○

DN9. Over the last 12 months, what was the maximum number of glasses of beer, wine and/or spirits that you drank in 24 hours?

	1-2	3-4	5-6	7-8	9-10	11-12	13-14	15-16	17-18	19 or more
**Maximum number of glasses per 24 hours:**	○	○	○	○	○	○	○	○	○	○

MEDICATION

Please provide as much information as you can about your medications. Thank you.

MED1. Do you currently take medication(s) prescribed by a doctor?

○ Yes ○ No ---> please go to MED3

MED2. Please list your doctor prescribed medications. *If possible, please copy the name of each medicine exactly as it appears on the package or container (include tablets, puffers, eye drops, creams, patches, suppositories, injections.)*

1

2

3

4

5

6

7

8

9

10

MED3. Please list your current medication which is purchased from a pharmacy, without a prescription.
If possible, please copy the name of each medicine exactly as it appears on the package or container (include vitamins & supplements, tablets, puffers, creams, patches, suppositories, aspirin etc)

1

2

3

BPMRI

MEDICAL HISTORY

We would be grateful if you could indicate if your doctor has ever told you that you had any of the following conditions. If you answer 'Yes', please write how old you were when you were first diagnosed (in the box to the right). Thank you for working through this list.

CANCER

MH1. Cancer ○ No ○ Yes

MH1a. If yes, what kind of cancer was it? If yes, age when first diagnosed

Cancer 1 years

Cancer 2 years

Cancer 3 years

MEDICAL HISTORY (continued)

CARDIOVASCULAR DISEASE			If yes, age when first diagnosed
**MH2. Angina**	○ No	○ Yes	☐☐ yrs
**MH3. Claudication** (problems with blood supply to your legs that causes pain on walking)	○ No	○ Yes	☐☐ yrs
**MH4. High blood pressure**	○ No	○ Yes	☐☐ yrs
If you are female, have you had high blood pressure or preeclampsia during pregnancy?	○ No	○ Yes	☐☐ yrs
**MH5. High cholesterol**	○ No	○ Yes	☐☐ yrs
**MH6. Implant of cardiac pacemaker**	○ No	○ Yes	☐☐ yrs
**MH7. Myocardial infarction / Heart attack**	○ No	○ Yes	☐☐ yrs
**MH8. Transient ischaemic attack (TIA)**	○ No	○ Yes	☐☐ yrs
**MH9. Stroke**	○ No	○ Yes	☐☐ yrs
**MH10. Carotid surgery (endarterectomy or stent)**	○ No	○ Yes	☐☐ yrs
**MH11. Coronary angioplasty or stent**	○ No	○ Yes	☐☐ yrs
**MH12. Coronary bypass**	○ No	○ Yes	☐☐ yrs

BPMRI

DIABETES

**MH13. Diabetes**	○ No	○ Yes	☐☐ yrs

If Yes, what kind of diabetes was it?
○ Type 1 diabetes (also known as insulin dependent diabetes)
○ Type 2 diabetes (also known as non-insulin dependent diabetes)

Were you told that your blood glucose level indicated diabetes?	○ No	○ Yes
Were you started on insulin treatment straight away?	○ No	○ Yes
If you are female, did you only have diabetes during pregnancy?	○ No	○ Yes

**MH14. Foot ulcers**	○ No	○ Yes	☐☐ yrs

ENDOCRINE DISEASE

**MH15. Osteroporosis**	○ No	○ Yes	☐☐ yrs
**MH16. Kidney disease**	○ No	○ Yes	☐☐ yrs
**MH17. Thyroid disease**	○ No	○ Yes	☐☐ yrs

MEDICAL HISTORY (continued)

NEUROLOGICAL CONDITIONS			If yes, age when first diagnosed
MH18. Alzheimer's disease	O No	O Yes	☐☐ yrs
MH19. Vascular dementia (Multi-infarct dementia)	O No	O Yes	☐☐ yrs
MH20. Parkinson's disease	O No	O Yes	☐☐ yrs
MH21. Attention Deficit (Hyperactivity) Disorder (AD(H)D)	O No	O Yes	☐☐ yrs
MH22. Anxiety disorder (including Post Traumatic Stress Disorder)	O No	O Yes	☐☐ yrs
MH23. Bipolar disorder	O No	O Yes	☐☐ yrs
MH24. Schizophrenia	O No	O Yes	☐☐ yrs
MH25. Epilepsy	O No	O Yes	☐☐ yrs
**ALLERGIES AND RESPIRATORY DISEASE**			
MH26. Asthma or bronchial asthma	O No	O Yes	☐☐ yrs
MH27. Eczema	O No	O Yes	☐☐ yrs
MH28. Bronchitis	O No	O Yes	☐☐ yrs
MH29. Chronic obstructive pulmonary disease (COPD)	O No	O Yes	☐☐ yrs
MH30. Hay fever or allergic rhinitis	O No	O Yes	☐☐ yrs
MH31. Pleurisy	O No	O Yes	☐☐ yrs
MH32. Pneumonia	O No	O Yes	☐☐ yrs
MH33. Sinusitis	O No	O Yes	☐☐ yrs
**SLEEP PROBLEMS**			
MH34. Narcolepsy	O No	O Yes	☐☐ yrs
MH35. Obstructive sleep apnoea	O No	O Yes	☐☐ yrs
**GASTROINTESTINAL DISORDERS**			
MH36. Stomach (gastric) or duodenal ulcer	O No	O Yes	☐☐ yrs
MH37. Colon cancer	O No	O Yes	☐☐ yrs

BPMRI

MEDICAL HISTORY (continued)

GASTROINTESTINAL DISORDERS (continued)

			If yes, age when first diagnosed
MH38. Colonic polyps	○ No	○ Yes	☐☐ yrs
MH39. Coeliac disease	○ No	○ Yes	☐☐ yrs
MH40. Gastro-oesophageal reflux disease	○ No	○ Yes	☐☐ yrs
MH41. Hiatus Hernia	○ No	○ Yes	☐☐ yrs
MH42. Crohn's disease	○ No	○ Yes	☐☐ yrs
MH43. Ulcerative colitis (or proctitis)	○ No	○ Yes	☐☐ yrs
MH44. Irritable bowel syndrome	○ No	○ Yes	☐☐ yrs
MH45. Diverticular disease	○ No	○ Yes	☐☐ yrs
MH46. Gallstones	○ No	○ Yes	☐☐ yrs
MH47. Haemorrhoids	○ No	○ Yes	☐☐ yrs

OTHER MEDICAL CONDITIONS

MH48. Chronic ear infection	○ No	○ Yes	☐☐ yrs
MH49. Ménière's Disease	○ No	○ Yes	☐☐ yrs
MH50. Trauma to the head or neck	○ No	○ Yes	☐☐ yrs
MH51. Anaemia	○ No	○ Yes	☐☐ yrs
MH52. Arthritis	○ No	○ Yes	☐☐ yrs
MH53. Migraine	○ No	○ Yes	☐☐ yrs
MH54. Headache	○ No	○ Yes	☐☐ yrs
MH55. Cirrhosis of the liver	○ No	○ Yes	☐☐ yrs
MH56. Fatty liver	○ No	○ Yes	☐☐ yrs
MH57. Poliomyelitis	○ No	○ Yes	☐☐ yrs
MH58. Urinary tract infection	○ No	○ Yes	☐☐ yrs

BPMRI

MEDICAL HISTORY (continued)

OTHER MEDICAL CONDITIONS (continued)

MH59. Other major medical condition(s) ○ No ○ Yes
If Yes, please specify below

If yes, age when first diagnosed

Condition 1 ____ years

Condition 2 ____ years

Condition 3 ____ years

Condition 4 ____ years

BPMRI

HEAD INJURY

It helps if we know about any history of head injury; even old injuries that occurred as a child.

HI1. Have you ever sustained any head injury involving loss of consciousness?

○ No ---> please go to EARS AND HEARING EH1 on page 12

○ Yes

HI2. If yes, when did the head injury occur?

DD MM YYYY

HI3. if you were hospitalised at the time, how long were you in hospital for?

days months years ○ Don't know

EARS AND HEARING

EH1 - HEARING

EH1a. Have you worked in a place where it was so noisy that you had to raise your voice to be heard by others?

- ○ No ---> please go to EH1c
- ○ Yes

EH1b. If yes, did you wear hearing protection?

○ Never ○ Occasionally ○ Frequently ○ Always

EH1c. Do you have a hearing impairment?

- ○ No ---> please go to EH2
- ○ Yes

EH1d. If you have a hearing impairment, does it affect your daily life and activities?

○ Not at all ○ Occasionally ○ Frequently ○ Constantly

EH1e. Do you use a hearing aid or other hearing device?

- ○ No
- ○ Hearing aid in one ear
- ○ Hearing aid in both ears
- ○ Cochlear implant
- ○ Bone Anchored Hearing Aid (BAHA)

BPMRI

EH2 - TINNITUS (INTERNAL SOUNDS)

EH2a. Do you experience tinnitus (sound in your ears and head) for <u>longer than 5 minutes</u>, which does not have an obvious cause?

- ○ No ---> please go to EH3 on page 13
- ○ Yes

EH2b. What is the frequency of your tinnitus?

○ Intermittent ○ Constant

EH2c. What is the nature of your tinnitus?

○ Ringing or hissing ○ Roaring ○ Pulsing ○ Other

EH2d. How does tinnitus affect your daily life and activities?

○ Not at all ○ Occasionally ○ Frequently ○ Constantly

EARS AND HEARING (continued)

EH3 - HYPERACUSIS (INTOLERANCE TO SOUND)

EH3a. Do you consider yourself sensitive or intolerant to everyday sounds?

- ○ No ---> please go to EH4
- ○ Yes

EH3b. Is it possible for you to concentrate on a task if it is not completely quiet around you?

○ No ○ Yes, most of the time ○ Yes

EH3c. Are you sensitive to any of these sounds? (select all that apply)

○ Noise ○ Paper ○ Talk ○ Music ○ Clatter ○ Mechanical and monotonous sounds ○ Other

EH3d. How do you feel when you are exposed to these sounds? (select all that apply)

○ Tense ○ Afraid ○ Pain ○ Angry ○ Vague ○ Irritated ○ Other

EH3e. If you are intolerant to some sound, how often does it affect your daily life and activities?

○ Not at all ○ Occasionally ○ Frequently ○ Constantly

EH4 - IMBALANCE

EH4a. Do you experience any imbalance or dizziness?

- ○ No ---> please go to EYES AND VISION EV1 on page 14
- ○ Yes

BPMRI

EH4b. What is the nature of your imbalance or dizziness? (Select all that apply)

- ○ Spinning or sensation of movement
- ○ Light-headedness
- ○ Unsteadiness on feet

EH4c. How often do you experience this imbalance or dizziness?

○ Daily ○ Weekly ○ Monthly ○ Less frequent than monthly

EH4d. How long do the specific episodes of imbalance or dizziness last?

- ○ Seconds to less than 2 minutes
- ○ 2 to 20 minutes
- ○ Over 20 minutes to hours
- ○ Hours to days

EH4e. How long do the after-effects of feeling unwell or off-colour last?

○ No after-effects ○ Minutes ○ Hours ○ Days

EARS AND HEARING (continued)

EH4 - IMBALANCE (continued)

EH4f. Do you suffer from any of the following symptoms for more than 20 minutes that you associate with your dizziness or imbalance? (Select all that apply.)

- ○ Fullness (blockage) in the ears
- ○ Tinnitus
- ○ Reduced hearing
- ○ Nausea
- ○ Vomiting

EH4g. Does your dizziness or imbalance occur when:

○ Sitting	○ Straining	○ Looking up to a high shelf
○ Walking	○ Bending down	○ Lying down and rolling over to one side
○ Sneezing	○ Hearing a loud noise	○ Standing up

EH4h. How often does your dizziness or imbalance affect your daily life and activities?

○ Not at all ○ Occasionally ○ Frequently ○ Constantly

EYES AND VISION

BPMRI

EV1. Do you wear glasses or contact lenses to correct your vision for seeing in the distance?

○ No ○ Yes

EV2. Do you have any other problems with your eyes or eyesight?

○ No ○ Yes

EV3. Has a doctor ever told you that you have any of the following problems with your eyes? (Select all that apply)

- ○ Diabetes related eye disease
- ○ Injury or trauma resulting in loss of vision
- ○ Macular degeneration
- ○ Glaucoma
- ○ Cataract
- ○ Other serious eye condition
- ○ None of the above
- ○ Don't know

RESPIRATORY

These questions ask about your breathing and the factors that affect how well you breathe.

R1 - WHEEZE

R1a. Has your chest ever made a wheezing or whistling sound? ○ No ○ Yes

R1b. If yes, in the last 12 months? ○ No ○ Yes

R2 - BREATHLESSNESS

R2a. Are you troubled by shortness of breath when hurrying on level ground or walking up a slight hill? ○ No ○ Yes

R2b. Do you get short of breath walking with other people your own age on level ground? ○ No ○ Yes

R2c. Do you have to stop for breath when walking at your own pace on level ground? ○ No ○ Yes

R2d. Do you ever get short of breath at rest? ○ No ○ Yes

BPMRI

R3 - COUGH

R3a. Do you usually cough first thing in the morning? ○ No ○ Yes

R3b. Do you usually cough during the day or at night? ○ No ○ Yes

---> <u>If you answered NO to BOTH questions R3a and R3b, please go on to question R4</u>

R3c. Do you cough like this on most days for as much as three months each year? ○ No ○ Yes

R4 - PHLEGM

R4a. Do you usually bring up phlegm from your chest first thing in the morning? ○ No ○ Yes

R4b. Do you usually bring up phlegm from your chest during the day or at night? ○ No ○ Yes
(If you answered Yes to R4a. or R4b, please answer R4c.)

---> <u>If you answered NO to BOTH questions R4a and R4b, please go on to question R5 on page 16</u>

R4c. Do you bring up phlegm like this on most days for as much as three months each year? ○ No ○ Yes

RESPIRATORY (continued)

R5 - CHEST TIGHTNESS

R5a. Have you ever felt tight in the chest? ○ No ○ Yes

R5b. If yes - In the last 12 months? ○ No ○ Yes

R6 - CARPET

R6a. Which rooms in your house are carpeted or have large rugs (more than 50% of the floor space)? (Select all that apply)

○ Your bedroom ○ Lounge ○ Living/Family ○ No carpet

R7 - PETS

R7a. Do you have a dog at home? (and where does it spend most of its time?)

○ Inside

○ Outside

○ No dog

R7b. Do you have a cat at home? (and where does it spend most of its time?)

○ Inside

○ Outside

○ No cat

R7c. Do you have a pet other than a cat or dog at home? (and where does it spend most of its time?)

○ Inside

○ Outside

○ No other pet

R7d. Please specify the type of other pet/s you have, if any:

Pet 1 [ ]

Pet 2 [ ]

Pet 3 [ ]

Pet 4 [ ]

Pet 5 [ ]

BPMRI

FOOD AND ALLERGIES

Another factor which can affect well-being is allergies. Please answer as fully as you can.

A1 - FOOD ALLERGIES

A1a. Have you ever been sick as a result of having eaten a particular food?

○ No ---> please go to A2 on page 18

○ Yes ------------------------------->

A1b. If yes, do you ever think that you have a food allergy?

○ No

○ Yes

A1c. Has anyone ever told you that you have a food allergy?

○ No ---> please go to A1e

○ Yes ------------------------------->

A1d. If yes, who told you this? Please select all that apply

○ GP

○ Dietician/other medical specialist

○ Naturopath/other complementary medicine practitioner

○ Other (such as a friend or relative)

A1e. In what way did the food make you feel ill when you ate it? (Select all that apply)

○ An itchy rash ○ Difficulty breathing ○ Flare up of eczema ○ Cough

○ Swelling of part of the body ○ Difficulty swallowing ○ Sore stomach ○ Vomiting

○ Hoarse voice ○ Felt like passing out ○ Other (please specify below)

A1f. How long after swallowing the food did you feel unwell?

○ Less than 2 hours ○ More than 2 hours

If more than 2 hours, how many hours was it? [ ][ ] hours

A1g. Which foods caused you to feel unwell (as described above)? (Please shade all that apply)

○ Eggs

○ Cow's milk/dairy products

○ Stone fruits

○ Shellfish (eg. prawn, oyster)

○ Peanuts

○ Finned fish (eg. snapper)

○ Soy

○ Wheat products (eg. bread)

○ Other (Please specify below)

BPMRI

FOOD AND ALLERGIES (continued)

A2 - ANAPHYLAXIS

A2a. Have you ever had a severe allergic reaction (also known as anaphylaxis) with light-headiness, a generalised rash, swelling of some part of the body, difficulty breathing, or loss of consciousness?

- ○ No ---> please go to SLEEP SL1
- ○ Yes

A2b. If yes, what did you react to? (Select all that apply)

- ○ Insect sting
- ○ Latex
- ○ Medication (such as antibiotic)
- ○ Food
- ○ Other (Please specify below)

A2c. Was this diagnosed by any of the following?

- ○ GP
- ○ Dietician/other medical specialist
- ○ Naturopath/other complementary medicine practitioner
- ○ Other (such as a friend or relative)

BPMRI

SLEEP

Below is a series of questions which ask about your sleep; a powerful determinant of well-being. Even if some of the questions seem similar, please answer each question independently and carefully.

SL1. How many hours of actual sleep do you usually get on a typical day, including naps?

[|] hours

SL2. Has your weight changed in the last 5 years?

- ○ Increased
- ○ Decreased
- ○ No change

SL3. Do you snore?

- ○ No ---> Please go to SL7 on page 19
- ○ Yes
- ○ Don't know

SL4. Has your snoring usually been:

- ○ About as loud as breathing
- ○ As loud as talking
- ○ Louder than talking
- ○ Very loud
- ○ Don't know

SLEEP (continued)

SL5. Does your snoring bother other people?

○ No ○ Yes

	Never or almost never	1-2 times per month	1- 2 times per week	3 -4 times per week	Almost every day	Don't Know
**SL6. How often do you snore?**	○	○	○	○	○	○
**SL7. How often have breathing pauses been noticed in your sleep?**	○	○	○	○	○	○
**SL8. Are you tired after sleeping?**	○	○	○	○	○	○
**SL9. Are you tired during wake time?**	○	○	○	○	○	○

SL10. Have you ever fallen asleep while driving?

○ No

○ Yes

SL11. How likely are you to doze off or fall asleep in the following situations, in contrast to feeling just tired? This refers to your usual way of life in recent times. Even if you have not done some of these things recently try to work out how they would have affected you.
Please select only one answer for each question.

	Never doze	Slight chance of dozing	Moderate chance of dozing	High chance of dozing
**SL11a. Sitting and reading**	○	○	○	○
**SL11b. Watching TV**	○	○	○	○
**SL11c. Sitting quietly in a public place (e.g. a theatre or meeting)**	○	○	○	○
**SL11d. As a passenger in a car for an hour without a break**	○	○	○	○
**SL11e. Lying down to rest in the afternoon when circumstances permit**	○	○	○	○
**SL11f. Sitting and talking to someone**	○	○	○	○
**SL11g. Sitting quietly after a lunch without alcohol**	○	○	○	○
**SL11h. In a car, while stopped for a few minutes in the traffic**	○	○	○	○

BPMRI

BACK PAIN

Back pain is one of the biggest contributors to loss of function, and days off work, so we need to understand this better.

SP1 - BACK PAIN BELIEFS

SP1. We are trying to find out what people think about low back trouble. Please indicate your general views towards back trouble, *even if you have never had any*. Please answer ALL statements.

	Completely disagree				Completely agree
**SP1a. There is no real treatment for back trouble**	O	O	O	O	O
**SP1b. Back trouble will eventually stop you from working**	O	O	O	O	O
**SP1c. Back trouble means periods of pain for the rest of one's life**	O	O	O	O	O
**SP1d. Doctors cannot do anything for back trouble**	O	O	O	O	O
**SP1e. A bad back should be exercised**	O	O	O	O	O
**SP1f. Back trouble makes everything in life worse**	O	O	O	O	O
**SP1g. Surgery is the most effective way to treat back trouble**	O	O	O	O	O
**SP1h. Back trouble may mean you end up in a wheelchair**	O	O	O	O	O
**SP1i. Alternative treatments are the answer to back trouble**	O	O	O	O	O
**SP1j. Back trouble means long periods of time off work**	O	O	O	O	O
**SP1k. Medication is the only way of relieving back trouble**	O	O	O	O	O
**SP1l. Once you have had back trouble there is always a weakness**	O	O	O	O	O
**SP1m. Back trouble must be rested**	O	O	O	O	O
**SP1n. Later in life back trouble gets progressively worse**	O	O	O	O	O

SP1o. Have your arms or legs been painful in the last month?

O No

O Yes

BPMRI

BACK PAIN (continued)

SP2 - NECK/SHOULDER PAIN

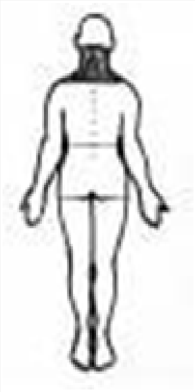

SP2a. Have you ever had neck/shoulder pain? (Anywhere in the shaded area in the picture)

○ No ---> please go to SP3a on page 22

○ Yes

SP2b. Has your neck/shoulder been painful at any time in the last month?

○ No ---> please go to SP3a on page 22

○ Yes

SP2c. How would you rate the neck/shoulder pain that you have had during the past week? Select one.

0	1	2	3	4	5	6	7	8	9	10
○	○	○	○	○	○	○	○	○	○	○
No pain										Pain as bad as it could be

SP2d. Has your present neck/shoulder pain lasted for more than 3 months continuously (it hurt more or less every day)

○ No

○ Yes

SP2e. Has your present neck/shoulder pain lasted for more than 3 months off and on? (it hurt at least once a week but not every day)

○ No

○ Yes

SP2f. Was your neck/shoulder pain initially caused by a specific injury or incident?

○ No

○ Yes

SP2g. Do you usually seek health professional advice or treatment for your neck/shoulder pain?

○ No

○ Yes

SP2h. Does your neck/shoulder pain usually interfere with your normal activities?

○ No

○ Yes

BPMRI

BACK PAIN (continued)

SP3 - MID BACK PAIN

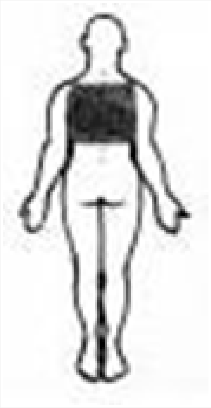

SP3a. Have you ever had mid back pain ? (Anywhere in the shaded area in the picture)

- ○ No ---> please go to SP4a on page 23
- ○ Yes

SP3b. Has your mid back been painful at any time in the last month?

- ○ No ---> please go to SP4a on page 23
- ○ Yes

SP3c. How would you rate the mid back pain pain that you have had during the past week? Select one.

0	1	2	3	4	5	6	7	8	9	10
○	○	○	○	○	○	○	○	○	○	○
No Pain										Pain as bad as it could be

SP3d. Has your present mid back pain lasted for more than 3 months continuously? (it hurt more or less every day)

- ○ No
- ○ Yes

SP3e. Has your present mid back pain lasted for more than 3 months off and on? (it hurt at least once a week but not every day)

- ○ No
- ○ Yes

SP3f. Was your mid back pain initially caused by a specific injury or incident?

- ○ No
- ○ Yes

SP3g. Do you usually seek health professional advice or treatment for your mid back pain?

- ○ No
- ○ Yes

SP3h. Does your mid back pain usually interfere with your normal activities?

- ○ No
- ○ Yes

BPMRI

BACK PAIN (continued)

SP4- LOW BACK PAIN

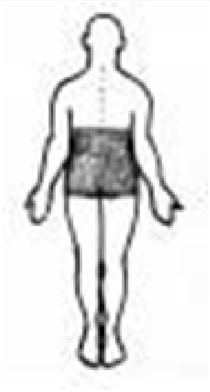

SP4a. Have you ever had low back pain (Anywhere in the shaded area in the picture)

- ○ No ---> please go to FAMILY HISTORY FH1 on page 28
- ○ Yes

SP4b. Has your low back been painful at any time in the last month?

- ○ No ---> please go to FAMILY HISTORY FH1 on page 28
- ○ Yes

SP4c. How would you rate the low back pain that you have had during the past week? Select one.

0	1	2	3	4	5	6	7	8	9	10
○	○	○	○	○	○	○	○	○	○	○
No Pain										Pain as bad as it could be

SP4d. Has your present low back pain lasted for more than 3 months continuously? (it hurt more or less every day)

- ○ No
- ○ Yes

SP4e. Has your present low back pain lasted for more than 3 months off and on? (it hurt at least once a week but not every day)

- ○ No
- ○ Yes

SP4f. Was your low back pain initially caused by a specific injury or incident?

- ○ No
- ○ Yes

SP4g. Do you usually seek health professional advice or treatment for your low back pain?

- ○ No
- ○ Yes

SP4h. Do you usually take medication to relieve your low back pain?

- ○ No
- ○ Yes

BPMRI

BACK PAIN (continued)

SP4 - LOW BACK PAIN (continued)

SP4i. Do you usually miss work due to your low back pain?

- ○ No
- ○ Yes

SP4j. Does your low back pain usually interfere with your normal activities?

- ○ No
- ○ Yes

SP4k. Does your low back pain usually interfere with recreational physical activities? (eg. sport, walking, cycling etc.)?

- ○ No
- ○ Yes

SP4l. At what age did you first get low back pain? ☐☐ years

BPMRI

SP5 - LOW BACK PAIN & EVERYDAY LIFE

The following questions have been designed to give information as to how your back pain has affected your ability to manage every day life. Please answer every section. Mark one box only in each section that most closely describes you today.

IF YOU HAVE NEVER HAD LOW BACK PAIN PLEASE GO TO FAMILY HISTORY FH1 ON PAGE 28

SP5a. Pain Intensity

- ○ I have no pain at the moment
- ○ The pain is very mild at the moment
- ○ The pain is moderate at the moment
- ○ The pain is fairly severe at the moment
- ○ The pain is very severe at the moment
- ○ The pain is the worst imaginable at the moment

SP5b. Personal Care (washing, dressing, etc.)

- ○ I can look after myself normally without causing extra pain
- ○ I can look after myself normally but it is very painful
- ○ It is painful to look after myself and I am slow and careful
- ○ I need some help but manage most of my personal care
- ○ I need help everyday in most aspects of self-care
- ○ I do not get dressed, wash with difficulty and stay in bed

BACK PAIN (continued)

SP5 - LOW BACK PAIN & EVERYDAY LIFE (continued)

SP5c. Lifting

- ○ I can lift heavy weights without extra pain
- ○ I can lift heavy weights but it gives extra pain
- ○ Pain prevents me from lifting heavy weights off the floor, but I can manage if they are conveniently positioned (e.g. table)
- ○ Pain prevents me from lifting heavy weights but I can manage light to medium weights if they are conveniently positioned
- ○ I can lift only very light weights
- ○ I cannot lift or carry anything at all

SP5d. Walking

- ○ Pain does not prevent me from walking any distance
- ○ Pain prevents me from walking more than 2 kilometres
- ○ Pain prevents me from walking more than 1 kilometre
- ○ Pain prevents me from walking more than 100 metres
- ○ I can only walk using a stick or crutches
- ○ I am in bed most of the time and have to crawl to the toilet

BPMRI

SP5e. Sitting

- ○ I can sit in any chair as long as I like
- ○ I can only sit in my favourite chair as long as I like
- ○ Pain prevents me from sitting for more than 1 hour
- ○ Pain prevents me from sitting for more than 1/2 an hour
- ○ Pain prevents me from sitting for more than 10 minutes
- ○ Pain prevents me from sitting at all

SP5f. Standing

- ○ I can stand as long as I want without extra pain
- ○ I can stand as long as I want but it gives me extra pain.
- ○ Pain prevents me from standing for more than 1 hour
- ○ Pain prevents me from standing for more than 1/2 an hour
- ○ Pain prevents me from standing for more than 10 minutes
- ○ Pain prevents me from standing at all

BACK PAIN (continued)

SP5 - LOW BACK PAIN & EVERYDAY LIFE (continued)

SP5g. Sleeping

- ○ My sleep is never disturbed by pain
- ○ My sleep is occasionally disturbed by pain
- ○ Because of pain I have less than 6 hours sleep
- ○ Because of pain I have less than 4 hours sleep
- ○ Because of pain I have less than 2 hours sleep
- ○ Pain prevents me from sleeping at all

SP5h. Sex life (if applicable)

- ○ My sex life is normal and causes no extra pain
- ○ My sex life is normal but causes some extra pain
- ○ My sex life is nearly normal but is very painful
- ○ My sex life is severely restricted by pain
- ○ My sex life is nearly absent because of pain
- ○ Pain prevents any sex life at all

BPMRI

SP5i. Social life

- ○ My social life is normal and gives me no extra pain
- ○ My social life is normal but increases the degree of pain
- ○ Pain has no significant effect on my social life apart from limiting my more energetic interests, e.g. sport
- ○ Pain has restricted my social life and I do not go out as often
- ○ Pain has restricted my social life to my home
- ○ I have no social life because of pain

SP5j. Travelling

- ○ I can travel anywhere without pain
- ○ I can travel anywhere but it gives me extra pain
- ○ Pain is bad but I manage journeys over two hours
- ○ Pain restricts me to journeys of less than one hour
- ○ Pain restricts me to short necessary journeys under 30 minutes
- ○ Pain prevents me from travelling except to receive treatment

BACK PAIN (continued)

SP6 - AFFECT ON BACK PAIN

Listed below are some of the things that people have told us about their pain.

SP6. For each statement below, please indicate any number from 0 to 6 to say how much physical activites such as bending, lifting, walking or driving, affect or would affect your back pain.

	Completely disagree 0	1	2	Unsure 3	4	5	Completely agree 6
**SP6a. My pain was caused by physical activity**	○	○	○	○	○	○	○
**SP6b. Physical activity makes my pain worse**	○	○	○	○	○	○	○
**SP6c. Physical activity might harm my back**	○	○	○	○	○	○	○
**SP6d. I should not do physical activities which (might) make my pain worse**	○	○	○	○	○	○	○
**SP6e. I cannot do physical activities which (might) make my pain worse**	○	○	○	○	○	○	○

The following statements are about how your normal work affects or would affect your back pain.

	Completely disagree 0	1	2	Unsure 3	4	5	Completely agree 6
**SP6f. My pain was caused by my work or by an accident at work**	○	○	○	○	○	○	○
**SP6g. My work aggravated my pain**	○	○	○	○	○	○	○
**SP6h. I have a claim for compensation for my pain**	○	○	○	○	○	○	○
**SP6i. My work is too heavy for me**	○	○	○	○	○	○	○
**SP6j. My work makes or would make my pain worse**	○	○	○	○	○	○	○
**SP6k. My work might harm my back**	○	○	○	○	○	○	○
**SP6l. I should not do my normal work with my present pain**	○	○	○	○	○	○	○
**SP6m. I cannot do my normal work with my present pain**	○	○	○	○	○	○	○
**SP6n. I cannot do my normal work until my pain is treated**	○	○	○	○	○	○	○
**SP6o. I do not think that I will be back to my normal work within 3 months**	○	○	○	○	○	○	○
**SP6p. I do not think that I will ever be able to go back to that work**	○	○	○	○	○	○	○

BPMRI

BACK PAIN (continued)

SP7. Individuals who experience pain have developed a number of ways to cope, or deal with their pain. These include saying things to themselves when they experience pain, or engaging in different activities. Below is a list of things that people have reported doing when they feel pain. For each activity, please indicate how much you engage in that activity when you feel pain.

When I feel pain….	Never do that						Always do that
**SP7a. It is terrible and I feel it is never going to get any better**	○	○	○	○	○	○	○
**SP7b. It is awful and I feel that it overwhelms me**	○	○	○	○	○	○	○
**SP7c. I feel my life isn't worth living**	○	○	○	○	○	○	○
**SP7d. I worry all the time about whether it will end**	○	○	○	○	○	○	○
**SP7e. I feel I can't stand it any more**	○	○	○	○	○	○	○
**SP7f. I feel like I can't go on**	○	○	○	○	○	○	○

SP8a. Have you retained a lawyer for your pain problem?

○ No ○ Yes

BPMRI

SP8b. How many days of work have you missed because of pain during the past 12 months?

○ 0 days ○ 1-2 days ○ 3-7 days ○ 8-14 days ○ 15-30 days

○ 31-60 days ○ 61-90 days ○ 91-180 days ○ 181-365 days

○ I do not work --> please go to FAMILY HISTORY FH1

SP8c. In your estimation, what are the chances that you will be able to work in six months?

0	1	2	3	4	5	6	7	8	9	10
○	○	○	○	○	○	○	○	○	○	○
No chance										Very large chance

FAMILY HISTORY

This next set of questions asks about your family history of medical problems. It helps us understand what contribution, if any, family history makes to our well-being.

FH1. Is your biological mother still alive?

○ I don't know if my biological mother is still alive

○ Yes ---> please go to FH1a --> **FH1a. How old is your mother?** [|] years

○ No ---> please go to FH1b --------------> **FH1b. How old was your mother when she died?** [|] years

FH1c. If no longer alive, cause of death if known

[| | | | | | | | | | | | | | | | |]

FAMILY HISTORY (continued)

FH2. Is your biological father still alive?

○ I don't know if my biological father is still alive

○ Yes ---> please go to FH2a -------------------------------------> **FH2a. How old is your father?** ☐☐ years

○ No ---> please go to FH2b ----------------> **FH2b. How old was your father when he died?** ☐☐ years

FH2c. If no longer alive, cause of death if known

FH3. Do you have any full (biological) brothers or sisters (same mother AND same father as you)?

○ No ---> please go to question FH7 on page 30

○ Yes -------------> **FH3a. How many brothers and sisters do you have?** ☐☐ brothers ☐☐ sisters

FH4a. How many brothers are still living?

☐☐ brothers living

Brother 1's age ☐☐ Brother 2's age ☐☐ Brother 3's age ☐☐ Brother 4's age ☐☐ Brother 5's age ☐☐ Brother 6's age ☐☐

FH4b. How many sisters are still living?

☐☐ sisters living

Sister 1's age ☐☐ Sister 2's age ☐☐ Sister 3's age ☐☐ Sister 4's age ☐☐ Sister 5's age ☐☐ Sister 6's age ☐☐

BPMRI

FH5. If any of your full (biological) brother(s) have died, please list ages when deceased:

		Cause of death if known
Brother 1 - Age when died	☐☐ years	
Brother 2 - Age when died	☐☐ years	
Brother 3 - Age when died	☐☐ years	
Brother 4 - Age when died	☐☐ years	

FH6. If any of your full (biological) sister(s) have died, please list ages when deceased:

		Cause of death if known
Sister 1 - Age when died	☐☐ years	
Sister 2 - Age when died	☐☐ years	
Sister 3 - Age when died	☐☐ years	
Sister 4 - Age when died	☐☐ years	

FAMILY HISTORY (continued)

Below is a table which asks about a history of a range of specific health conditions in your immediate biological family (mother, father, sisters and brothers). Please take the time to indicate, for each family member and for each condition, if that person had that condition or not. If you do not know, please check 'Don't know'. Any other information you can give us would also be helpful (e.g. about conditions we have not listed, but which you feel are important).

FH7 Have any of your following family members ever had any of the following conditions?

	Biological mother	Biological father	Any of your biological sisters	Any of your biological brothers
**FH7a. Asthma**	○ Yes ○ No ○ Don't know	○ Yes ○ No ○ Don't know	○ Yes ○ No ○ Don't know	○ Yes ○ No ○ Don't know
**FH7b. Diabetes**	○ Yes ○ No ○ Don't know	○ Yes ○ No ○ Don't know	○ Yes ○ No ○ Don't know	○ Yes ○ No ○ Don't know
**FH7c. If Yes to FH7b, was this only during pregnancy?**	○ Yes ○ No ○ Don't know		○ Yes ○ No ○ Don't know	
**FH7d. Hay Fever - seasonal**	○ Yes ○ No ○ Don't know	○ Yes ○ No ○ Don't know	○ Yes ○ No ○ Don't know	○ Yes ○ No ○ Don't know
**FH7e. Hay Fever - all year round**	○ Yes ○ No ○ Don't know	○ Yes ○ No ○ Don't know	○ Yes ○ No ○ Don't know	○ Yes ○ No ○ Don't know
**FH7f. Hearing loss**	○ Yes ○ No ○ Don't know	○ Yes ○ No ○ Don't know	○ Yes ○ No ○ Don't know	○ Yes ○ No ○ Don't know
**FH7fg. High blood pressure**	○ Yes ○ No ○ Don't know	○ Yes ○ No ○ Don't know	○ Yes ○ No ○ Don't know	○ Yes ○ No ○ Don't know
**FH7h. Myocardial Infarction (Heart Attack)**	○ Yes ○ No ○ Don't know	○ Yes ○ No ○ Don't know	○ Yes ○ No ○ Don't know	○ Yes ○ No ○ Don't know
**FH7i. Stroke**	○ Yes ○ No ○ Don't know	○ Yes ○ No ○ Don't know	○ Yes ○ No ○ Don't know	○ Yes ○ No ○ Don't know
**FH7j. Glaucoma**	○ Yes ○ No ○ Don't know	○ Yes ○ No ○ Don't know	○ Yes ○ No ○ Don't know	○ Yes ○ No ○ Don't know

BPMRI

FAMILY HISTORY (continued)

FH7 (continued). Have any of your following family members ever had any of the following conditions?

	Biological mother	Biological father	Any of your biological sisters	Any of your biological brothers
**FH7k. Macular degeneration**	O Yes<br>O No<br>O Don't know	O Yes<br>O No<br>O Don't know	O Yes<br>O No<br>O Don't know	O Yes<br>O No<br>O Don't know
**FH7l. Cancer**	O Yes<br>O No<br>O Don't know	O Yes<br>O No<br>O Don't know	O Yes<br>O No<br>O Don't know	O Yes<br>O No<br>O Don't know

FH7m. If yes, type of cancer

Biological mother's type of cancer [ ]

O Don't know mother's cancer type

Biological father's type of cancer [ ]

O Don't know father's cancer type

Biological sister 1's type of cancer [ ]

O Don't know sisters 1's cancer type

Biological sister 2's type of cancer [ ]

O Don't know sister 2's cancer type

Biological sister 3's type of cancer [ ]

O Don't know sister 2's cancer type

Biological brother 1's type of cancer [ ]

O Don't know brother 1's cancer type

Biological brother 2's type of cancer [ ]

O Don't know brother 2's cancer type

Biological brother 3's type of cancer [ ]

O Don't know brother 3's cancer type

FH8. Are there any conditions or illnesses which run in your family (other than those mentioned above)?

O Don't know

O No

O Yes, please specify below

[ ]

[ ]

BPMRI

Questionnaire ID 1

MOOD AND WELL-BEING

Now we would like to ask some questions about your general mood and well-being. We realise that some of these questions may seem very personal, but all information that you provide us is helpful. As before, even if some questions seem remarkably similar, we need to ask you each and every one. So, please answer them carefully and independently.

MD1. Please read each statement and select a number 0,1,2 or 3 which indicates how much the statement applied to you over the past week. There are no right or wrong answers. Do not spend too much time on any statement.

		Did not apply to me at all **0**	Applied to me to some degree, or some of the time **1**	Applied to me a considerable degree, or a good part of time **2**	Applied to me very much, or most of the time **3**
**MD1a.**	**I found it hard to wind down**	○	○	○	○
**MD1b.**	**I was aware of dryness of my mouth**	○	○	○	○
**MD1c.**	**I couldn't seem to experience any positive feeling at all**	○	○	○	○
**MD1d.**	**I experienced breathing difficulty** (eg. excessively rapid breathing, breathlessness in the absence of physical exertion.)	○	○	○	○
**MD1e.**	**I found it difficult to work up the initiative to do things**	○	○	○	○
**MD1f.**	**I tended to over-react to situations**	○	○	○	○
**MD1g.**	**I experienced trembling (eg. in the hands)**	○	○	○	○
**MD1h.**	**I felt that I was using a lot of nervous energy**	○	○	○	○
**MD1i.**	**I was worried about situations in which I might panic and make a fool of myself**	○	○	○	○
**MD1j.**	**I felt that I had nothing to look forward to**	○	○	○	○
**MD1k.**	**I found myself getting agitated**	○	○	○	○
**MD1l.**	**I found it difficult to relax**	○	○	○	○
**MD1m.**	**I felt down-hearted and blue**	○	○	○	○
**MD1n.**	**I was intolerant of anything that kept me from getting on with what I was doing**	○	○	○	○
**MD1o.**	**I felt I was close to panic**	○	○	○	○
**MD1p.**	**I was unable to become enthusiastic about anything**	○	○	○	○
**MD1q.**	**I felt I wasn't worth much as a person**	○	○	○	○
**MD1r.**	**I felt that I was rather touchy**	○	○	○	○
**MD1s.**	**I was aware of the action of my heart in the absence of physical exertion** (eg. sense of heart rate increase, heart missing a beat)	○	○	○	○
**MD1t.**	**I felt scared without any good reason**	○	○	○	○
**MD1u.**	**I felt that life was meaningless**	○	○	○	○

BPMRI

MOOD AND WELL-BEING (continued)

MD2. Over the <u>last 2 weeks</u>, how often have you been bothered by any of the following problems? Read each item carefully and fill in your response.

		Not at all	Several days	More than half the day	Nearly every day
**MD2a.**	**Little interest or pleasure in doing things**	○	○	○	○
**MD2b.**	**Feeling down, depressed, or hopeless**	○	○	○	○
**MD2c.**	**Trouble falling asleep, staying asleep, or sleeping too much**	○	○	○	○
**MD2d.**	**Feeling tired or having little energy**	○	○	○	○
**MD2e.**	**Poor appetite or overeating**	○	○	○	○
**MD2f.**	**Feeling bad about yourself, feeling that you are a failure, or feeling that you have let yourself or your family down**	○	○	○	○
**MD2g.**	**Trouble concentrating on things such as reading the newspaper or watching television**	○	○	○	○
**MD2h.**	**Moving or speaking so slowly that other people could have noticed; or being so fidgety or restless that you have been moving around a lot more than usual**	○	○	○	○
**MD2i.**	**Thinking that you would be better off dead or that you want to hurt yourself in some way**	○	○	○	○

MD3. If you have noted any problem in questions MD1-MD2, how difficult have these problems made it for you to do your work, take care of things at home, or get along with other people?

○ Not difficult at all ○ Somewhat difficult ○ Very difficult ○ Extremely difficult

BPMRI

DEPRESSION

We are particularly interested to learn whether you have ever experienced depression.

DP1. Have you ever been told by a doctor that you have depression?

○ No ---> please go to PHYSICAL ACTIVITY PA1 on page 34

○ Yes

DP2. Please state your age when you were first told that you had depression ☐☐ years

DP3. Have you ever been given advice or treatment for your depression?

○ No ---> please go to PHYSICAL ACTIVITY PA1 on page 34

○ Yes

DP4. What kind of advice or treatment were you given for your depression? (Please answer <u>**each**</u> question)

Tablets	○ Yes	○ No
Exercise	○ Yes	○ No
Psychological treatment or counselling	○ Yes	○ No
Electro convulsive therapy (ECT)	○ Yes	○ No
Other (please specify below)	○ Yes	○ No

☐☐☐☐☐☐☐☐☐☐☐☐☐☐☐☐☐☐☐☐☐☐

DEPRESSION (continued)

DP5. Please state your age when you were first given advice or treatment for depression ☐☐ years

DP6. What kind of advice or treatment are you following now for depression? (Please answer **each** question)

None	○ Yes	○ No
Tablets	○ Yes	○ No
Exercise	○ Yes	○ No
Psychological treatment or counselling	○ Yes	○ No
Electro convulsive therapy (ECT)	○ Yes	○ No
Other (please specify below)	○ Yes	○ No

PHYSICAL ACTIVITY

Earlier, we asked you a few, brief questions about your general physical activity. Here, we would like to go into this in more detail. We are interested in finding out about the kinds of physical activities that people do as part of their everyday lives. The questions will ask you about the time you spent being physically active in the last 7 days. Please answer each question, even if you do not consider yourself to be an active person. Please think about the activities you do at work, as part of your house and yard work, to get from place to place, and in your spare time for recreation, exercise or sport.

*Think about all the **vigorous** physical activities that you did in the **last 7 days**. **Vigorous** physical activities refer to activities that take hard physical effort and make you breathe much harder than normal. Think only about those physical activities that you did for **at least 10 minutes at a time**.*

PA1. During the last 7 days, on how many days did you do vigorous physical activities like heavy lifting, digging, aerobics, or fast bicycling?

○ No vigorous activities ---> please go to PA3

☐ days per week

PA2. How much time did you usually spend doing vigorous physical activities on one of those days?

☐☐ hours per day ☐☐ minutes per day

○ Don't know/Not sure

*Think about all the **moderate** physical activities that you did in the **last 7 days**. **Moderate** activities refer to activities that take moderate physical effort and make you breathe somewhat harder than normal. Think only about those physical activities that you did for at **least 10 minutes at a time**.*

PA3. During the last 7 days, on how many days did you do moderate physical activities like carrying light loads, bicycling at a regular pace, or doubles tennis? Do not include walking.

○ No moderate activities ---> Please go to PA5 on page 35

☐ days per week

BPMRI

PHYSICAL ACTIVITY (continued)

PA4. How much time did you usually spend doing moderate physical activities on one of those days?

[ ][ ] hours per day [ ][ ] minutes per day

○ Don't know/Not sure

*Think about the time you spent **walking** in the **last 7 days**. This includes at work and at home, walking to travel from place to place, and any other walking that you might do solely for recreation, sport, exercise, or leisure.*

PA5. During the last 7 days, on how many days did you walk for at least 10 minutes at a time?

○ No walking ---> please go to PA7

[ ] days per week

PA6. How much time did you usually spend walking on one of those days?

[ ][ ] hours per day [ ][ ] minutes per day

○ Don't know/Not sure

*This question is about the time you spent **sitting on weekdays** during **the last 7 days.** Include time spent at work, at home, while doing course work and during leisure time. This may include time spent sitting at a desk, visiting friends, reading, or sitting to watch television.*

PA7. During the last 7 days, how much time did you spend sitting on a week day?

[ ][ ] hours per day [ ][ ] minutes per day

○ Don't know/Not sure

BPMRI

INFORMATION TECHNOLOGY

This next section asks about your use of information technology (mobile phone, computer, internet etc).

IT1a. For approximately how many years have you been using a mobile phone at least once per week to make or receive calls?

○ Never used a mobile phone ---> please go to IT2a

○ 1 year or less ○ 2-4 years ○ 5-8 years ○ More than 8 years

IT1b. Over the last 3 months, on average how much time per week did you spend making or receiving calls on a mobile phone?

[ ][ ] hours per day [ ][ ] minutes per day

IT2a. Do you have access to a TV/video/DVD at home?

○ No ---> please go to IT3a on page 36

○ Yes

Questionnaire ID 1

IT2b. In the last month, how often did you watch TV or videos or DVDs?

- ○ Not at all ---> please go to IT3
- ○ Once a month ○ Once a week ○ Two or three times a week ○ Daily

IT2c. In the last month, for how long did you usually watch TV or videos or DVDs each day?

- ○ Less than 30 minutes ○ 30-60 minutes ○ 1-2 hours ○ 2-5 hours ○ More than 5 hours

IT3a. Have you ever used a computer?

- ○ No ---> please go to COMMUNITY AND VALUES CV1 on page 38
- ○ Yes

IT3b. About what age were you when you started using a computer? ☐☐ years

The next few questions ask you about computer use at work only

IT4a. In the last month, how often did you use a computer at work?

- ○ Not at all ---> please go to IT5a on page 37
- ○ Do not work ---> please go to IT5a on page 37
- ○ Once a month ○ Once a week ○ Two or three times a week ○ Daily

IT4b. In the last month, for how long did you usually use a computer at work each time?

- ○ Less than 30 minutes ○ 30-60 minutes ○ 1-2 hours ○ 2-5 hours ○ More than 5 hours

In the last month, how often did you do the following activities on a computer at work?
Please select one answer for each statement.

		Not at all	Once a month	Once a week	2- 3 times a week	Daily
IT4c.	Play games	○	○	○	○	○
IT4d.	Use/create multi-media (e.g. pictures and music)	○	○	○	○	○
IT4e.	Use documents/spreadsheets	○	○	○	○	○
IT4f.	Surf the internet	○	○	○	○	○
IT4g.	Send/receive emails	○	○	○	○	○
IT4h.	Chat room	○	○	○	○	○
IT4i.	Other activities e.g. learning programs, work databases	○	○	○	○	○

IT4j. Thinking about the last seven days, in total how many hours have you spent using a computer at work? ☐☐ hours

BPMRI

INFORMATION TECHNOLOGY (continued)

The next few questions ask you about computer use <u>at home only.</u>
This includes using a computer at a friend's home)

IT5a. Do you have access to a computer at home?

- ○ No ---> please go to IT6a on page 38
- ○ Yes

IT5b. Do you have internet access at home?

- ○ No
- ○ Yes

IT5c. How many desktop computers do you have at home? ☐☐

IT5d. How many laptop computers do you have at home? ☐☐

IT5e. In the last month, how often did you use a computer at home?

- ○ Not at all ---> please go to IT6a on page 38
- ○ Once a month ○ Once a week ○ Two or three times a week ○ Daily

BPMRI

IT5f. In the last month, for how long did you usually use a computer at home each time?

○ Less than 30 minutes ○ 30-60 minutes ○ 1-2 hours ○ 2-5 hours ○ More than 5 hours

In the last month, how often did you do the following activities on a computer <u>at home</u>?
Please select one answer for each statement.

		Not at all	Once a month	Once a week	2- 3 times a week	Daily
IT5g.	Play games	○	○	○	○	○
IT5h.	Use/create multi-media (e.g. pictures and music)	○	○	○	○	○
IT5i.	Use documents/spreadsheets	○	○	○	○	○
IT5j.	Surf the internet	○	○	○	○	○
IT5k.	Send/receive emails	○	○	○	○	○
IT5l.	Chat room	○	○	○	○	○
IT5m.	Other activities e.g. learning programs, databases	○	○	○	○	○

IT5n. Thinking about the <u>last seven days</u>, in total how many hours have you spent using a computer at home or at a friend's home? ☐☐ hours

Questionnaire ID 1

INFORMATION TECHNOLOGY (continued)

IT6. How do you feel generally when you use computers anywhere
(please select one answer for each statement)

		Strongly Disgree	Moderately Disgree	Slightly Disagree	Neutral	Slightly Agree	Moderately Agree	Strongly Agree
IT6a.	Computers do not scare me at all.	○	○	○	○	○	○	○
IT6b.	I do not feel anxious when other people talk about computers.	○	○	○	○	○	○	○
IT6c.	I get butterflies in the stomach when I think of trying to use a computer.	○	○	○	○	○	○	○
IT6d.	I would feel comfortable working with a computer.	○	○	○	○	○	○	○
IT6e.	Computers make me feel uneasy and confused.	○	○	○	○	○	○	○
IT6f.	I'm no good with computers.	○	○	○	○	○	○	○
IT6g.	Generally I would feel OK about trying a new problem on the computer.	○	○	○	○	○	○	○
IT6h.	I'm not the type to do well with computers	○	○	○	○	○	○	○
IT6i.	I think using a computer would be very hard for me.	○	○	○	○	○	○	○
IT6j.	I have a lot of confidence in my ability when it comes to working with computers.	○	○	○	○	○	○	○

COMMUNITY VALUES

It seems that who you know and where you live can influence your health and wellbeing.
We are interested to know how you feel about where you live and the people around you.

		No, not at all	No, not much	Yes, somewhat/ to some degree	Yes, definitely
CV1a.	Do you feel safe walking down your street after dark?	○	○	○	○
CV1b.	Do you agree that most people can be trusted?	○	○	○	○
CV1c.	If someone's car breaks down outside your house, do you invite them into your house to use the phone?	○	○	○	○
CV1d.	Can you get help from friends when you need it?	○	○	○	○
CV1e.	Does your area have a reputation for being a safe place?	○	○	○	○
CV1f.	If you were caring for a child and needed to go out for a while, would you ask a neighbour for help?	○	○	○	○
CV1g.	Have you visited a neighbour in the past week?	○	○	○	○
CV1h.	Does your local community feel like home?	○	○	○	○
CV1i.	When you go shopping in your local area, are you likely to run into friends and acquaintances?	○	○	○	○

BPMRI

COMMUNITY VALUES (continued)

What kind of values do you uphold as an Australian family? We are interested to know what life values are important to you. For every item, please select the answer that best suits your family.

	No, not at all	No, not much	Yes, somewhat/ to some degree	Yes, definitely
**CV2a. Having money for nice things**	O	O	O	O
**CV2b. Being popular with lots of people**	O	O	O	O
**CV2c. Having a high status job**	O	O	O	O
**CV2d. Playing an active role in the community**	O	O	O	O
**CV2e. Helping others who are less well off**	O	O	O	O
**CV2f. Looking after our planet**	O	O	O	O
**CV2g. Saving or investing for the future**	O	O	O	O
**CV2h. Having a financial plan for the future**	O	O	O	O
**CV2i. Having a fit and healthy lifestyle**	O	O	O	O
**CV2j. Having close personal relationships**	O	O	O	O

BPMRI

You have reached the end of the questionnaire!

Thank you very much for your time and effort!

We appreciate that there were many questions and realise that it will have taken you quite some time to complete the survey. We are grateful to you for your patience in helping us with this important study.

If you have any queries about any of the questions - for example, you were not sure how to answer a question - please ask the BHAS survey staff for clarification when you visit for your appointment.

This page intentionally left blank

BPMRI
