# Supplementary material for: Prevalence and patterns of multimorbidity in Australian baby boomers: the Busselton healthy ageing study
Source: BMC Public Health. 2021 Aug 11;21:1539. doi: 10.1186/s12889-021-11578-y (PMC8359115; doi:10.1186/s12889-021-11578-y)
Supplement: Supplementary file 2 — Additional file 2. Supplementary File 2. Statistical Supplement. Latent Class Analysis method. [file 12889_2021_11578_MOESM2_ESM.docx]

**Statistical Supplement – Latent Class Analysis**

Two complementary analytical methods were used to explore multi-morbidity patterns. The common and direct exploratory method is to calculate the observed absolute prevalence of pairs and triplets but this needs to be adjusted for the likelihood of having the combination by chance alone and hence the observed/expected prevalence ratio is used. With individuals potentially having any number and combination of the 21 possible conditions there will be thousands of observed disease combinations. To better understand disease patterns it is therefore necessary to use statistical techniques that divide the population into distinct disease profiles.

Latent Class Analysis (LCA) is a maximum-likelihood model-based statistical technique that is fit for the purpose of partitioning a population into a fixed number of latent classes with distinct disease patterns and has been increasingly used and recommended for identifying patterns of multimorbidity (see references (18, 21, 36). In LCA the presence/absence of the 21 conditions is modelled as a function of membership of a fixed number of latent classes. True class membership is unknown. Instead, the model produces the best fitting estimates of the proportion of the population in each class and sets of conditional probabilities that someone in a particular class will be positive for a certain condition. Further the model produces estimates of the probability that each individual belongs to each latent class and these are used to assign each sample individual to a best-fit class (based on their highest probability of membership). The disease profiles and characteristics of each assigned class are then interpreted and examined.

The appropriate number of latent classes is determined by fitting a sequence of LCA models (with 2, 3, 4, etc classes) and the relative fit of the models assessed using the change in likelihood ratio statistic, Akaike Information Criterion (AIC), and the adjusted Bayesian Information Criterion (BIC) statistics. Model interpretability is also considered, e.g. distinguishability of each class from the others on the basis of the condition probabilities, size (no class with very few individuals), and the possibility of assigning a meaningful label to each class. Based on these considerations, the model with four latent classes was considered appropriate for our analysis of 21 conditions among 5029 individuals.
