# Supplementary material for: Prevalence and patterns of multimorbidity in Australian baby boomers: the Busselton healthy ageing study
Source: BMC Public Health. 2021 Aug 11;21:1539. doi: 10.1186/s12889-021-11578-y (PMC8359115; doi:10.1186/s12889-021-11578-y)
Supplement: Supplementary file 3 — Additional file 3. Supplementary Table S1. Mean, median and maximum number of conditions by gender and demographic groups. [file 12889_2021_11578_MOESM3_ESM.docx]

**Supplementary Table S1. Mean, median and maximum number of conditions by gender and demographic groups.**

|  | | **Men** | | | | |  | **Women** | | | | |
| --- | --- | --- | --- | --- | --- | --- | --- | --- | --- | --- | --- | --- |
| **Demography** |  | **N** | **Mean** | **Std Dev** | **Median** | **Maximum** |  | **N** | **Mean** | **Std Dev** | **Median** | **Maximum** |
| ***Age (years)*** | 45-49 | 222 | 2.18 | 1.38 | 2.00 | 7 |  | 270 | 1.96 | 1.47 | 2.00 | 9 |
|  | 50-54 | 547 | 2.35 | 1.54 | 2.00 | 10 |  | 669 | 2.31 | 1.66 | 2.00 | 10 |
|  | 55-59 | 567 | 2.60 | 1.82 | 2.00 | 10 |  | 713 | 2.69 | 1.75 | 2.00 | 9 |
|  | 60-64 | 611 | 3.04 | 1.87 | 3.00 | 13 |  | 750 | 3.16 | 2.03 | 3.00 | 12 |
|  | 65-69 | 326 | 3.44 | 2.00 | 3.00 | 11 |  | 354 | 3.50 | 1.99 | 3.00 | 10 |
|  |  |  |  |  |  |  |  |  |  |  |  |  |
| ***Marital status*** | Single | 97 | 2.86 | 1.71 | 3.00 | 8 |  | 111 | 3.06 | 1.96 | 3.00 | 10 |
|  | Married | 1791 | 2.72 | 1.82 | 2.00 | 13 |  | 1985 | 2.65 | 1.82 | 2.00 | 11 |
|  | Widowed | 20 | 2.90 | 1.99 | 2.00 | 7 |  | 105 | 3.34 | 2.06 | 3.00 | 9 |
|  | Divorced | 131 | 3.19 | 1.87 | 3.00 | 9 |  | 273 | 3.19 | 2.07 | 3.00 | 12 |
|  | Separated | 44 | 2.48 | 1.47 | 2.00 | 6 |  | 84 | 2.63 | 1.80 | 2.00 | 7 |
|  | Defacto | 188 | 2.60 | 1.67 | 2.00 | 7 |  | 197 | 2.84 | 1.88 | 3.00 | 10 |
|  |  |  |  |  |  |  |  |  |  |  |  |  |
| ***Education level*** | Primary school | 34 | 3.00 | 1.74 | 3.00 | 7 |  | 33 | 3.73 | 1.53 | 4.00 | 7 |
|  | Secondary school | 1140 | 2.76 | 1.85 | 2.00 | 13 |  | 1326 | 2.81 | 1.90 | 3.00 | 12 |
|  | Technical school | 671 | 2.76 | 1.78 | 2.00 | 10 |  | 847 | 2.78 | 1.92 | 2.00 | 10 |
|  | University | 424 | 2.63 | 1.73 | 2.00 | 11 |  | 547 | 2.58 | 1.76 | 2.00 | 9 |
|  |  |  |  |  |  |  |  |  |  |  |  |  |
| ***Employment*** | In paid employment | 1649 | 2.49 | 1.60 | 2.00 | 9 |  | 1604 | 2.42 | 1.69 | 2.00 | 10 |
|  | Retired | 446 | 3.24 | 1.99 | 3.00 | 11 |  | 701 | 3.15 | 1.96 | 3.00 | 10 |
|  | Home or family duties | 15 | 2.07 | 2.02 | 2.00 | 7 |  | 208 | 2.86 | 1.84 | 3.00 | 9 |
|  | Unable to work due to sickness or disability | 78 | 4.69 | 2.15 | 4.00 | 10 |  | 93 | 4.77 | 2.38 | 4.00 | 12 |
|  | Voluntary work | 11 | 4.64 | 3.59 | 4.00 | 13 |  | 66 | 3.14 | 2.12 | 3.00 | 9 |
|  | Unemployed | 44 | 3.07 | 1.98 | 3.00 | 10 |  | 55 | 3.38 | 1.83 | 3.00 | 7 |
|  | Other | 26 | 3.12 | 1.92 | 3.00 | 7 |  | 30 | 2.60 | 1.33 | 3.00 | 6 |
|  |  |  |  |  |  |  |  |  |  |  |  |  |
| ***Household income*** | Less than $20,000 | 85 | 3.91 | 2.31 | 3.00 | 13 |  | 218 | 3.56 | 2.08 | 3.00 | 12 |
|  | $20,001 - $40,000 | 298 | 3.14 | 2.06 | 3.00 | 10 |  | 531 | 3.19 | 2.12 | 3.00 | 10 |
|  | $40,001 to $60,000 | 385 | 2.92 | 1.82 | 3.00 | 11 |  | 450 | 2.79 | 1.76 | 3.00 | 10 |
|  | $60,001 to $80,000 | 309 | 2.73 | 1.83 | 2.00 | 9 |  | 350 | 2.61 | 1.80 | 2.00 | 10 |
|  | $80,001 to $100,000 | 303 | 2.45 | 1.64 | 2.00 | 10 |  | 298 | 2.36 | 1.58 | 2.00 | 8 |
|  | More than $100,000 | 650 | 2.42 | 1.54 | 2.00 | 8 |  | 490 | 2.32 | 1.60 | 2.00 | 8 |
|  | Not disclosed | 234 | 2.80 | 1.77 | 3.00 | 10 |  | 404 | 2.68 | 1.90 | 2.00 | 11 |
|  |  |  |  |  |  |  |  |  |  |  |  |  |
