# Supplementary material for: Prevalence and patterns of multimorbidity in Australian baby boomers: the Busselton healthy ageing study
Source: BMC Public Health. 2021 Aug 11;21:1539. doi: 10.1186/s12889-021-11578-y (PMC8359115; doi:10.1186/s12889-021-11578-y)
Supplement: Supplementary file 4 — Additional file 4. Supplementary Table S2. Relationship between number of conditions and participant characteristics. [file 12889_2021_11578_MOESM4_ESM.docx]

Supplementary Table S2. Relationship between number of conditions and participant characteristics.

| **Characteristic** | **Coefficient Estimate** | **Standard Error** | **T Value** | **P Value** |
| --- | --- | --- | --- | --- |
| *Intercept* | -5.392042 | 0.299405 | -18.01 | <.0001 |
| Sex ^ *(Female)* | 0.409946 | 0.056214 | 7.29 | <.0001 |
| Age *(years)* | 0.067534 | 0.004302 | 15.7 | <.0001 |
| Waist circumference *(cm)* | 0.04009 | 0.002005 | 20 | <.0001 |
| Tobacco smoker^^ |  |  |  |  |
| Current 15 cigarettes per day | 0.735523 | 0.110206 | 6.67 | <.0001 |
| Current <15 cigarettes per day | 0.37452 | 0.11972 | 3.13 | 0.0018 |
| Former smoker | 0.066501 | 0.051109 | 1.3 | 0.1933 |
|  |  |  |  |  |
| Moderate/vigorous physical activity  (mean total hours per week) | -0.004892 | 0.002109 | -2.32 | 0.0204 |
|  |  |  |  |  |
| Mean number of first degree relatives with: |  |  |  |  |
| Asthma | 0.260017 | 0.039253 | 6.62 | <.0001 |
| Diabetes | 0.111971 | 0.038561 | 2.9 | 0.0037 |
| Myocardial infarct | 0.081744 | 0.036028 | 2.27 | 0.0233 |
| Cancer (any) | 0.107953 | 0.028273 | 3.82 | 0.0001 |
|  |  |  |  |  |

Reference categories – ^male, ^^never smoked tobacco
