# Supplementary material for: Prevalence and patterns of multimorbidity in Australian baby boomers: the Busselton healthy ageing study
Source: BMC Public Health. 2021 Aug 11;21:1539. doi: 10.1186/s12889-021-11578-y (PMC8359115; doi:10.1186/s12889-021-11578-y)
Supplement: Supplementary file 5 — Additional file 5 Supplementary Table S3. All triplets of conditions with prevalence > 1.5% and observed/expected (O/E) ratio > 1.5 (and p value< 0.0001). [file 12889_2021_11578_MOESM5_ESM.docx]

**Supplementary** Table S3. All triplets of conditions with prevalence >1.5% and observed/expected (O/E) ratio >1.5 (and p value<0.0001).

| **Co-occurring triplets of conditions** | | | | | **Prevalence (%)** | **O/E** |
| --- | --- | --- | --- | --- | --- | --- |
| Arthritis | + | Low back pain | + | Depression–anxiety | 2.33 | 5.31 |
| Arthritis | + | Low back pain | + | Bronchitis | 1.67 | 4.43 |
| Arthritis | + | Low back pain | + | Bowel disease | 2.01 | 4.04 |
| Asthma | + | COPD | + | Atopy | 1.75 | 3.95 |
| Bowel disease | + | Low back pain | + | Depression–anxiety | 1.59 | 3.92 |
| Arthritis | + | Asthma | + | Bronchitis | 1.73 | 2.75 |
| Arthritis | + | Bowel disease | + | Eye condition | 2.23 | 2.62 |
| Arthritis | + | Depression–anxiety | + | Bronchitis | 2.35 | 2.59 |
| Arthritis | + | Bowel disease | + | Asthma | 2.09 | 2.52 |
| Arthritis | + | Bowel disease | + | Depression–anxiety | 3 | 2.51 |
| Arthritis | + | Bowel disease | + | Bronchitis | 2.51 | 2.43 |
| Arthritis | + | Depression–anxiety | + | Eye condition | 1.71 | 2.28 |
| Bowel disease | + | Depression–anxiety | + | Bronchitis | 1.67 | 1.99 |
| Arthritis | + | Low back pain | + | Atopy | 2.25 | 1.93 |
| Depression–anxiety | + | Low back pain | + | Atopy | 1.81 | 1.91 |
| Asthma | + | Bronchitis | + | Atopy | 2.55 | 1.87 |
| Arthritis | + | Asthma | + | Atopy | 3.62 | 1.87 |
| Liver disease | + | Depression–anxiety | + | Bronchitis | 1.53 | 1.84 |
| Arthritis | + | Liver disease | + | Bronchitis | 1.83 | 1.79 |
| Bowel disease | + | Asthma | + | Atopy | 3.04 | 1.69 |
| OSA | + | Liver disease | + | Atopy | 1.69 | 1.67 |
| Arthritis | + | Liver disease | + | Depression–anxiety | 1.93 | 1.63 |
| Arthritis | + | Liver disease | + | Bowel disease | 2.17 | 1.61 |
| Arthritis | + | Bowel disease | + | Atopy | 4.97 | 1.56 |
| Asthma | + | Eye condition | + | Atopy | 1.73 | 1.54 |
| Depression–anxiety | + | Asthma | + | Atopy | 2.39 | 1.51 |
| Depression–anxiety | + | Bowel disease | + | Liver disease | 1.65 | 1.5 |
