# Supplementary material for: Prevalence and patterns of multimorbidity in Australian baby boomers: the Busselton healthy ageing study
Source: BMC Public Health. 2021 Aug 11;21:1539. doi: 10.1186/s12889-021-11578-y (PMC8359115; doi:10.1186/s12889-021-11578-y)
Supplement: Supplementary file 6 — Additional file 6. Supplementary Table S4. Morbidity patterns and number of conditions. Number of participants with each number of conditions and percentage within each morbidity pattern. [file 12889_2021_11578_MOESM6_ESM.docx]

**Supplementary** Table S4. Morbidity patterns and number of conditions. Number of participants with each number of conditions and percentage within each morbidity pattern.

|  | Morbidity Patterns* | | | |  |
| --- | --- | --- | --- | --- | --- |
| Number (%) conditions | Class 1 | Class 2 | Class 3 | Class 4 | Total |
| 0 | 364  (100) | 0 | 0 | 0 | 364 |
| 1 | 1001  (98.4) | 16  (1.6) | 0 | 0 | 1017 |
| 2 | 1069  (91.2) | 94  (8.0) | 9  (0.8) | 0 | 1172 |
| 3 | 745  (73.9) | 144  (14.3) | 115  (11.4) | 4  (0.4) | 1008 |
| 4 | 285  43.6 | 145  22.2 | 194  (29.7) | 29  (4.4) | 653 |
| 5 | 70  (17.2) | 78  (19.2) | 196  (48.2) | 63  (15.5) | 407 |
| 6 | 7  (3.1) | 41  (18.4) | 100  (44.8) | 75  (33.6) | 223 |
| 7 | 0 | 10  (11) | 49  (53.8) | 32  (35.2) | 91 |
| 8 | 0 | 1  (1.8) | 22  (38.6) | 34  (59.6) | 57 |
| 9 | 0 | 0 | 2  (13.3) | 13  (86.7) | 15 |
| 10 | 0 | 0 | 4  (22.2) | 14  (77.8) | 18 |
| 11 | 0 | 0 | 0 | 2  (100) | 2 |
| 12 | 0 | 0 | 0 | 1  (100) | 1 |
| 13 | 0 | 0 | 0 | 1  (100) | 1 |
| Total | 3541  (70.4) | 529  (10.5) | 691  (13.7) | 268  (5.3) | 5029  (100) |

* Latent class analysis.
